# Large-Scale Synthesis of Covalent Organic Frameworks: Challenges and Opportunities

**DOI:** 10.3390/membranes13080696

**Published:** 2023-07-27

**Authors:** Harsh Vardhan, Grace Rummer, Angela Deng, Shengqian Ma

**Affiliations:** 1 Department of Chemistry and Fermentation Sciences, Appalachian State University, 525 Rivers Street, Boone, NC 28608, USA; 2 Department of Chemistry, University of North Texas, Denton, TX 76203, USA

**Keywords:** covalent organic frameworks (COFs), dynamic covalent chemistry, topologies, activation, synthetic and designing principles

## Abstract

Connecting organic building blocks by covalent bonds to design porous crystalline networks has led to covalent organic frameworks (COFs), consequently transferring the flexibility of dynamic linkages from discrete architectures to extended structures. By virtue of the library of organic building blocks and the diversity of dynamic linkages and topologies, COFs have emerged as a novel field of organic materials that propose a platform for tailor-made complex structural design. Progress over the past two decades in the design, synthesis, and functional exploration of COFs in diverse applications successively established these frameworks in materials chemistry. The large-scale synthesis of COFs with uniform structures and properties is of profound importance for commercialization and industrial applications; however, this is in its infancy at present. An innovative designing and synthetic approaches have paved novel ways to address future hurdles. This review article highlights the fundamental of COFs, including designing principles, coupling reactions, topologies, structural diversity, synthetic strategies, characterization, growth mechanism, and activation aspects of COFs. Finally, the major challenges and future trends for large-scale COF fabrication are outlined.

## 1. Introduction

**Figure 1:**
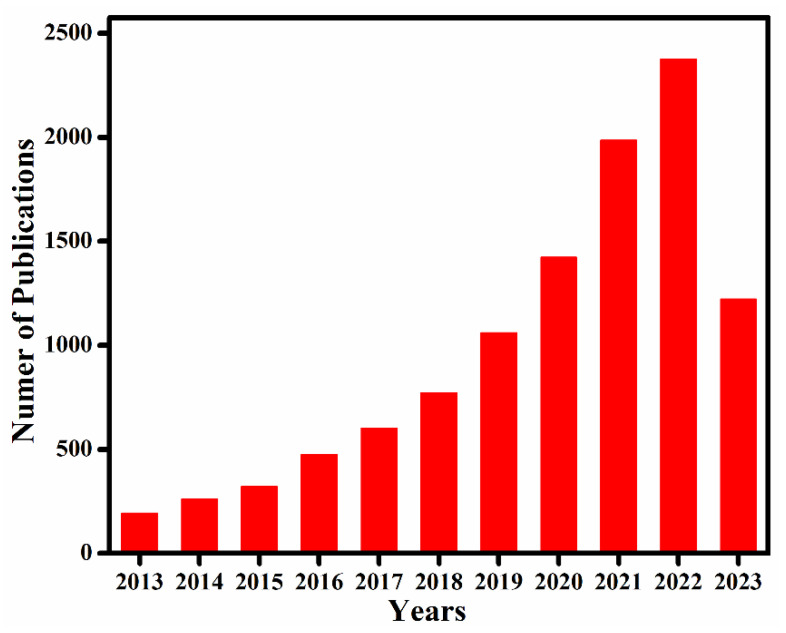
Number of published papers on covalent organic frameworks in the last ten years. Data obtained from Web of Science using the keyword “covalent organic frameworks”.

The covalent chemistry of organic molecules has matured throughout the 20th century and at the core of many important advances in science. The synthesis of chemicals, polymers, and pharmaceuticals via the modification of organic molecules through stable covalent bonds have fundamentally changed our way of life [1,2]. The fabrication of well-defined macromolecules with precise primary and high-order structures, as found in biological polymers, such as proteins and enzymes, is challenging [3,4]. Biological polymers with a well-defined structure amalgamate the chemistry of covalent bonds to understand primary-order chain structure and intermolecular force of attraction to shape up the high-order morphology [5,6]. Inspired by biological polymer systems, the role of covalent bonds and non-covalent interactions in achieving synthetic primary and high-order structure with predesigned functionalities is significant to address fundamental challenges, thus expanding chemistry of hierarchical structures. Covalent organic frameworks (COFs) are porous and crystalline polymers discovered nearly two-decades ago by stitching molecular building blocks together through the covalent bonds and noncovalent interactions in the polymerization systems [7,8,9,10]. As stated by Nobel laureate Roald Hoffmann: “Organic chemists are masterful at exercising control in the zero dimensions. One subculture of organic chemists has learned to exercise control in one dimension. These are polymer chemists, the chain builders…. But in two or three dimensions, it’s a synthetic wasteland” [11]. In 2005, Yaghi and co-workers made a breakthrough in successfully connecting boronic acid- and catechol-based building blocks to an extended porous crystalline boroxine- and boronic-ester-linked COFs using the principle of dynamic covalent chemistry [10]. Since then, the exponential rate of frameworks grown via the emergence of novel dynamic linkages, such as imine, hydrazone, imide, azine, and β-ketoenamine, is extraordinary (Figure 1) [12,13,14,15].

A crystalline framework with exclusive conformation and morphology generates a confined molecular space (pores) with the accessibility of atoms facilitating host–guest interactions. Furthermore, the porosity and structure stability are of profound importance to expand functional development via post-synthetic modification [16,17,18]. Pore surface engineering permits the steric and electronic tunability of the pore environment by using the principles of organic and organometallic chemistry. Based on these properties, COFs are at the forefront in heterogeneous catalysis [19,20,21], environmental remediation [22,23,24,25,26,27], energy storage [28,29,30], biomedical applications [31,32,33], and other applications [34,35,36]. In recent times, a number of notable reviews have summarized different COF perspectives, especially in the area of reticular chemistry [37,38,39], covalent chemistry [9,40,41], pore surface engineering [16,17,18,42], and diverse applications [43,44,45,46] (Table 1), but to date, fundamental aspects of the large-scale synthesis of COFs have not been reviewed. In this review, we cover recent advances in the fundamental concepts and summarizes coupling chemistry, design principles, topologies, growth mechanism, synthetic and activation strategies, and scale-up fabrication and predict the future directions from multiple perspectives to boost commercialization. 

## 2. Coupling Chemistry

Coupling chemistry refers to a variety of organic reactions where two fragments are stitched together. COFs are built from organic building blocks via reversible condensation reactions. This micro-reversibility prevents the formation of disordered amorphous kinetic products and bolsters thermodynamically stable crystalline covalent networks. Furthermore, the reversibility in bond formation imparts self-healing and error-correction during crystallization. Throughout the reversible covalent bond formation and extension, if any bond formation happens in an undesired direction, the system can repair it through a back reaction and bond reformation, thereby supporting crystalline thermodynamic products with the lowest free energy. However, the condition for reversible covalent bond formation can be achieved only at a very-high temperature and pressure owing to the higher covalent bond energies (50–100 kcal mol^−1^). At lower temperatures, kinetically controlled disordered polymeric products are seen to dominate; thus, it is difficult to construct the ordered covalent network solids under ambient reaction conditions. Moreover, the desired thermodynamic reaction pathways demand a very-high activation energy. 

**Figure 2:**
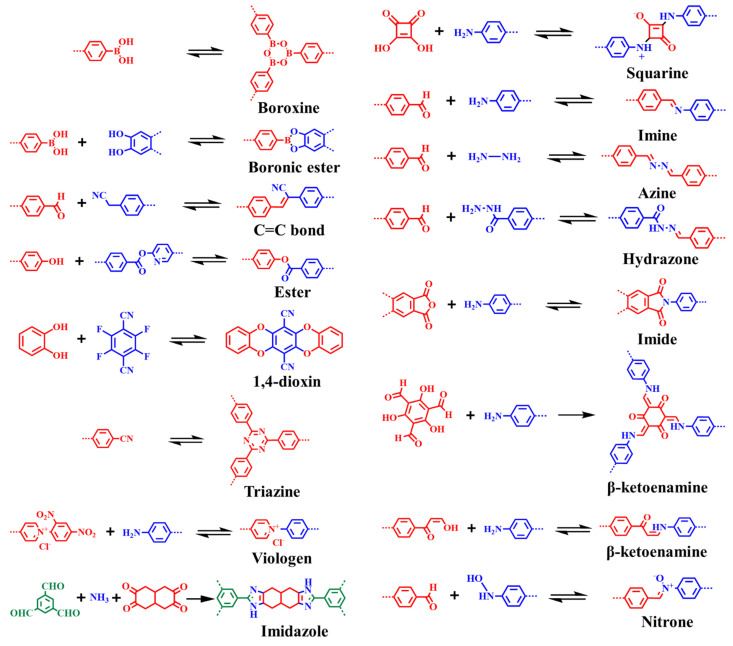
Widely accepted reactions for the formation of COFs.

**Figure 3:**
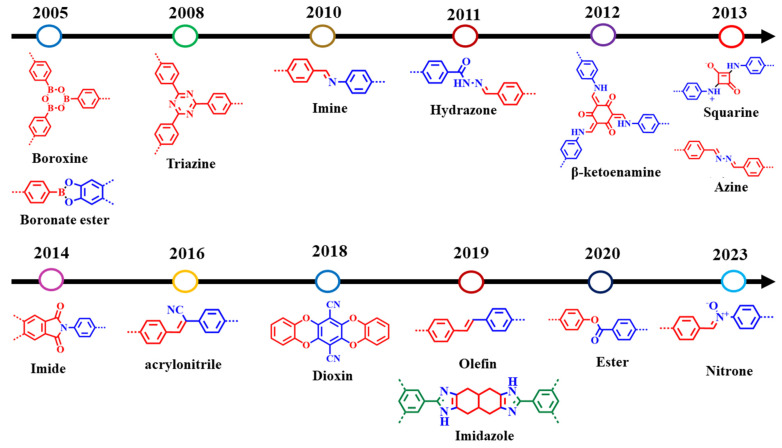
Timeline of various dynamic linkages for COF formation.

The reversible nature of coupling reaction permits the formation of crystalline structures. For instance, the first framework synthesized by the condensation of boronic-acid- and catechol-based monomers forms five-membered boronic ester rings as the linkage between the building blocks. Furthermore, the self-condensation of boronic-ester-based building blocks generates boroxines [10]. As boronic esters and boroxines are prone to hydrolysis, a range of stable dynamic linkages were achieved (Figure 2). In 2009, Yaghi and co-workers reported imine-linked frameworks by the condensation of primary amine and aldehyde [104]. Since then, hydrazone, azine, triazine, imide, squaraine, and other linkages were achieved to enhance frameworks thermal and chemical stability (Figure 3 and Table 2) [54,56,105,106,107,108,109,110,111,112]. To further enhance acidic/basic stability, Banerjee’s research group reported β-ketoenamine-linked COFs by the condensation reaction of 1,3,5-triformylphloroglucinol and primary amine, followed by enol–keto tautomerization [113]. In contrast to reversible coupling reactions, the irreversible nuclear aromatic substitution reaction (S_N_Ar) between ortho-difluoro benzene and catechol building blocks generate 1,4-dioxine-linked COF-316 and COF-318 with good thermal and chemical stability [114]. Furthermore, chemically stable microporous polyarylether-based JUC-505 (1.68 nm) and mesoporous polyarylether-based JUC-506 (2.84 nm) were synthesized by the substitution reaction of a triangular building unit, 2,3,6,7,10,11-hexahydroxytriphenylene with tetrafluoroterephthalonitrile and 2,3,6,7-tetrafluoroanthraquinone, respectively [115]. The scope of dynamic linkages and building blocks in COFs structures and functionalities can be understood by the Debus–Radziszewski reaction. Wang and co-workers reported ultrastable imidazole-linked LZU-510, LZU-506, LZU-508, and LZU-512 by the covalent assemblies of *C*_3_-symmetric aldehyde, tertbutylpyrene tetraone, and ammonium acetate at 150 °C in dioxane/mesitylene. Considering the excellent chemical stability of imidazole-linked COFs in acid (9M HCl), base (9M NaOH), and water, DMF opens novel avenue for post-synthetic modification. For instance, LZU-501 underwent a one-step N-alkylation reaction under NaH/THF/65 °C afford ethyl-, benzyl-, pyridine-, and morpholine-modified LZU-501 with good crystallinity and porosity [116,117]. Recently, ester- and nitrone-linked COFs constructed by connecting tetrakis(4-hydroxyphenyl)ethylene with di(pyridine-2-yl)terephthalate and terephthalaldehyde with N,N′,N″,N‴-(ethene-1,1,2,2-tetrayltetrakis(benzene-4,1-diyl)tetrakis-(hydroxylamine), respectively, were also comprehended to advance novel coupling reactions in frameworks chemistry [118,119].

Apart from the conventional [1 + 1] two-component approach, a multiple-component reaction strategy offers multiformity and structurally diverse skeletons. The orthogonal reaction usually involves reversible formations of covalent bonds, which is an imperative prerequisite for the construction of crystalline frameworks. Zhao and co-workers reported orthogonal reaction strategy to construct binary NTU-COF-1 and ternary NTU-COF-2 by reversible formation of two types of covalent bonds [120]. Two-dimensional NTU-COF-1 was synthesized by the formation of a boroxine ring and imine functionalities through the solvothermal condensation reaction of 1,3,5-tris(4-aminophenyl)benzene and 4-formylphenylboronic acid. However, NTU-COF-2 was constructed from a three-component condensation reaction, including 4-formylphenylboronic acid, 1,3,5-tris(4-aminophenyl)benzene, and 2,3,6,7,10,11-hexahydroxytriphenylene, through the formation of C_2_O_2_B boronate ring and imine functionalities. Due to tunability and multiple functionalities, NTU-COF-2 displayed a high hydrogen uptake capacity of 1.55 wt%, which is higher than those of COF-1 (1.28 wt%), COF-5 (0.85 wt%), and COF-102 (1.29 wt%) and comparable to that of CTF-1 (1.55 wt%). 

## 3. Design Principles and Topologies

**Figure 4:**
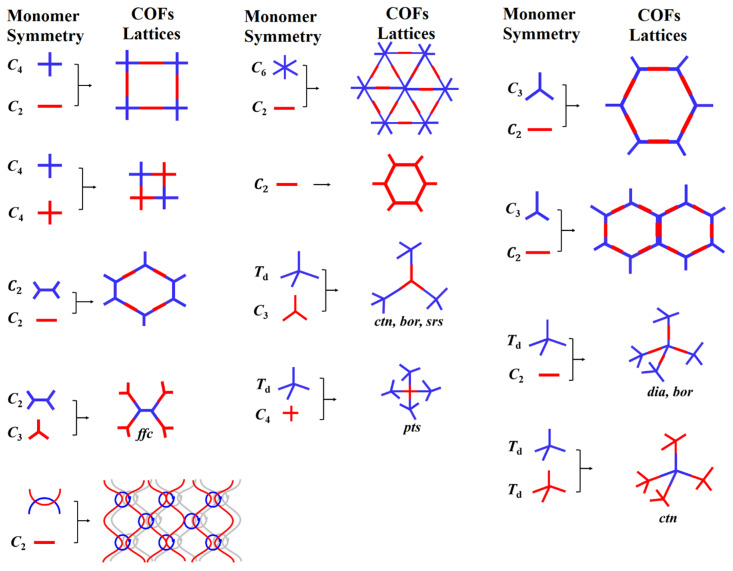
Two-dimensional and three-dimensional COF topological diagrams.

Linking organic building blocks toward topology-directed framework growth occurs in concurrence with the geometry of building blocks. A high-order crystalline structure with unique conformation and morphology relies on the directional nature of covalent bonds. A covalent bond is a chemical bond made by sharing electrons between atoms. Organic molecule synthesis exhibits the full benefits of the directionality of covalent bonds, as exemplified by the synthesis of vitamin B_12_ [121,122]. Organic building blocks with rigid backbone and distribution of reactive sites in a distinct geometry ensure directional bonding. Furthermore, building blocks of particular symmetricity and point group guide spatial orientation and determine the relative position of the repeated units (chain growth direction) that firmly follow the predesigned topology. The principle of directional bonding is established in the construction of transition metal-based discrete architectures, such as metalla-rectangle and metalla-prism [123,124]. The stitching of symmetrical organic building blocks via dynamic linkages affords 2D atomic layers with specific topology. The periodic growth of a 2D layer under the influence of noncovalent interactions inherently generates discrete nanopores. This bestows a well-defined hierarchical system with spatial lattice orientation and crystallinity through controlled interlayer interactions. The high-order structure with an accessible one-dimension open channels are primarily dependent on the geometry of building blocks often referred to as bottom-up approach. The topology of COFs with varying pore shapes and sizes can be understood to have different permutations and combinations of building blocks. For instance, the combination of [*C*_2_ + *C*_2_ + *C*_2_], [*C*_3_ + *C*_2_], and [*C*_3_ + *C*_3_] symmetric organic building blocks form hexagonal COFs with different pore sizes and stacking patterns [125,126]. However, [*C*_4_ + *C*_4_] and [*C*_4_ + *C*_2_] linkers afford a tetragonal framework of varying pore size (Figure 4). Notably, either the [*C*_2_ + *C*_2_ + *C*_2_] or [*C*_4_ + *C*_4_] combinations result in COFs with micropores (<2 nm), whereas [*C*_3_ + *C*_2_], [*C*_3_ + *C*_3_], and [*C*_4_ + *C*_2_] yield mesoporous COFs (2–50 nm). The symmetricity, point group, and structural features of organic building blocks bolster interlayer (π–π) interactions and facilitates COF topology. 

Apart from 2D COFs, a 3D COF design entails at least one tetrahedral (*T*_d_) or orthogonal geometry of building blocks to enable the extension of the backbone into a 3D network. For instance, the stitching of tetrahedral or orthogonal nodes with *C*_1_, *C*_2_, *C*_3_, *C*_4_, and tetragonal organic building blocks produce 3D COFs (Figure 4) [45,95]. The ctn network can be obtained by linking [*T*_d_ + *C*_3_] and [*T*_d_ + *T*_d_] symmetric building blocks, whereas a bor network can be obtained by [*T*_d_ + *C*_3_]. The *pts* network can be formed by either [*T*_d_ + *C*_2_] or [*T*_d_ + *C*_4_], in which the *C*_2_ or *C*_4_ symmetric unit exhibits four reactive sites. In addition, the dia and srs networks can be formed by [*T*_d_ + *C*_2_] and [*T*_d_ + *C*_3_], respectively. The dia net among ctn, bor, srs, rra, pts, she, scu, bcu, fjh, and other networks constitute the largest family of 3D COFs due to the diversity and library of organic building blocks, which in turn emphasize the pivotal role of novel linkers and dynamic linkages. Notably, the mechanistic study and crystallization complications due to imbalance between strong covalent bonds and reversible linkages are still unexplored. Furthermore, 3D COFs suffer from lower porosity due to multifold interpenetration, which is one of the critical tasks in terms of design and construction perspectives. These fundamentals restrict the library of frameworks with unique dynamic linkages and an electronic and steric environment. 

## 4. Pore Shapes and Structures

**Figure 5:**
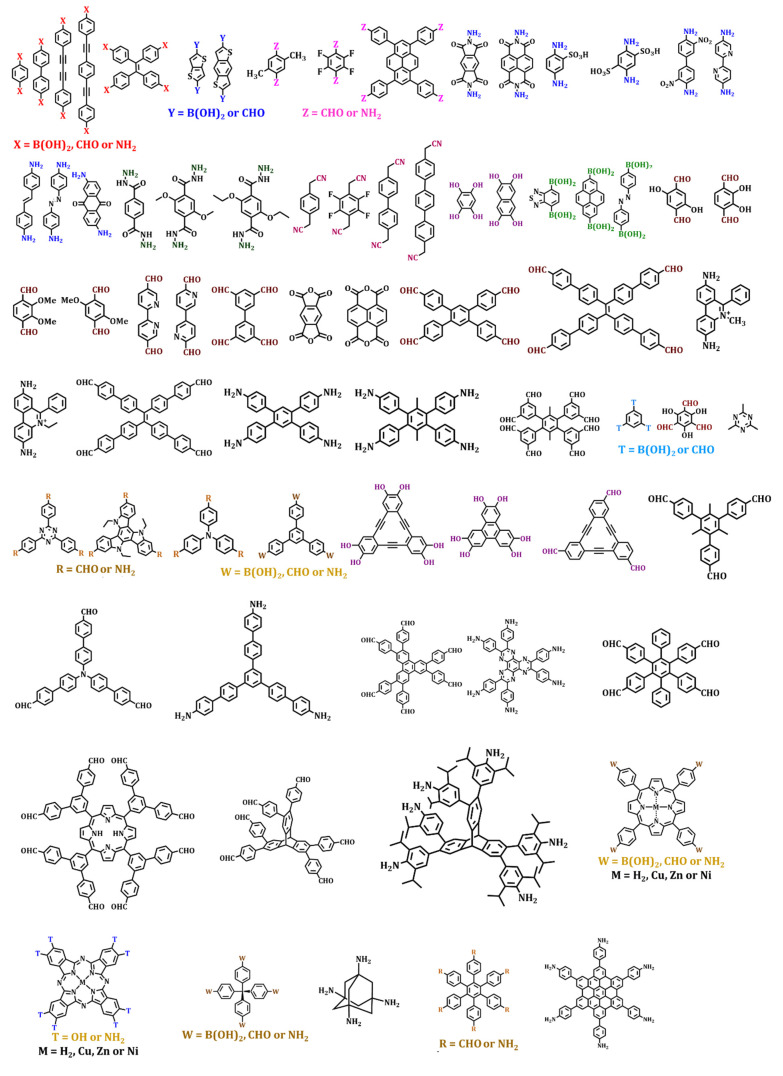
Organic building blocks with different geometries and reactive groups.

**Figure 6:**
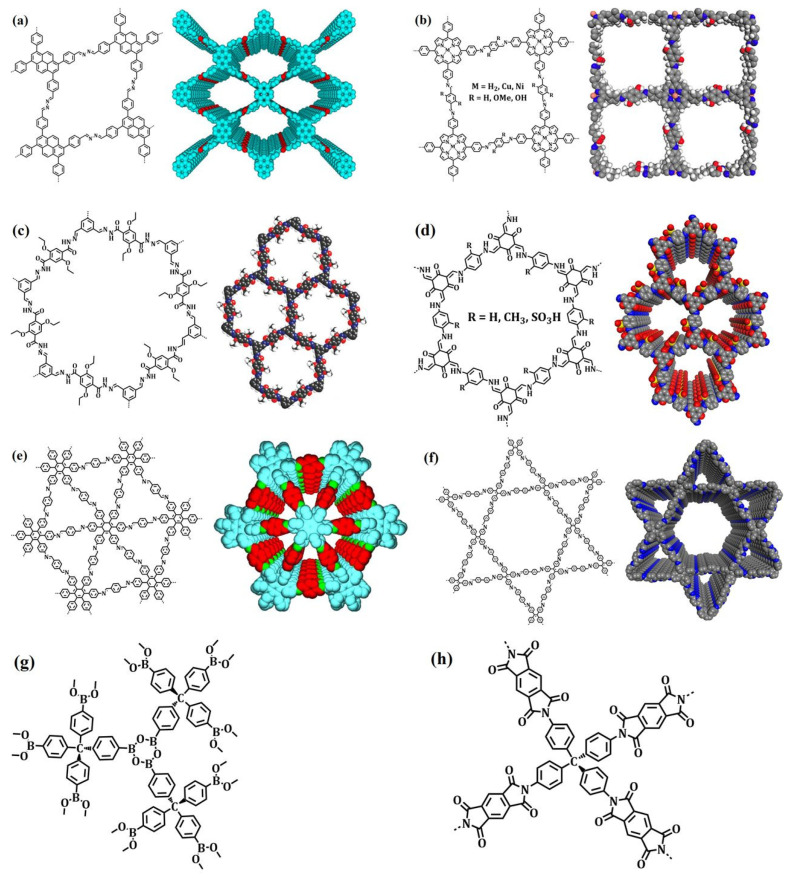
Skelton design and stacking structure of COFs for (**a**) rhombic, azine-linked Py-azine COFs; (**b**) tetragonal imine-linked MP-COFs; (**c**) hexagonal hydrazone-linked COFs; (**d**) hexagonal β-ketoenamine TpPa COFs; (**e**) triangular imine-linked HPB-COFs; (**f**) heteropore imine-linked COFs; (**g**) 3D boroxine-linked COF-102; and (**h**) 3D imide-linked PI-COF-5. Reprinted with permission from [105,106,138,162] (Copyright © American Chemical Society).

Numerous building blocks with unique geometrical and structural features enable the design of COFs with varying pore shapes and sizes. This permits the frameworks’ topological diversity via the bottom-up approach. Furthermore, building blocks of varying sizes and functionalities have been extensively explored to tune the electronic and steric environment of 2D frameworks (Figure 5 and Figure 6). On the other hand, 3D COFs exhibit a limited structural diversity due to the restricted number of monomers with *T*_d_ or orthogonal nodes and inadequate dynamic linkages. Nonetheless, the expansion of 3D COFs with conventional coupling reactions is still a major challenge.

### 4.1. Rhombic COFs

Rhombic-shaped COFs are designed by connecting *C*_3_- and *C*_2_-symmetric building blocks in either a [*C*_2_ + *C*_2_] or [*C*_3_ + *C*_3_] manner. The chemically and thermally stable imine-linked, azine-linked, and olefin-linked COFs, such as Py-Azine, Py-2,3-DHPh, Py-3,3′-BPyPh, Py-2PE, sp^2^c-COF-2, sp^2^c-COF-3, and HAT-NTBA-COF, possess rhombic lattices with pore sizes larger than 1.5 nm and smaller than 3.0 nm [106,111,127,128,129]. For instance, Por-sp^2^c-COF was synthesized by the reversible Knoevenagel condensation reaction of 5,10,15,20-tetrakis(4-benzaldehyde)porphyrin and 1,4-phenylenediacetonitrile in *o*-DCB/DBU at 80 °C for 3 days. The framework exhibited a good thermal (250 °C) and chemical stability even under harsh conditions, such as 9M HCl and 9M NaOH. Notably, the porphyrin-based rhombic COF displayed a moderate Brunauer–Emmett–Teller (BET) surface area (689 m^2^/g) and was employed as a metal-free heterogeneous photocatalyst for the visible-light-induced aerobic oxidation of amines to imines. In addition, fully *p*-conjugated 2D crystalline sp^2^c-COF, sp^2^c-COF-2, and sp^2^c-COF-3 frameworks were constructed by the olefin-linkage of tetrakis(4-formylphenyl)pyrene with 1,4-phenylenediacetonitrile, 2,2′-(biphenyl-4,4′-diyl)diacetonitrile, and 2,2′-([1,1′:4′,1″-terphenyl]-4,4″-diyl)diacetonitrile, respectively [127]. These rhombic, porous frameworks exhibited excellent thermal and chemical stability outcomes in acidic, basic, protic, and aprotic solutions. Notably, sp^2^c-COF-2 and sp^2^c-COF were stable in air conditions for over a year and exhibited a BET surface area as high as 581 m^2^/g and 302 m^2^/g, respectively, which is in close agreement with the as-synthesized frameworks (sp^2^c-COF, 613 m^2^/g; and sp^2^c-COF-2, 322 m^2^/g). This is important for the large-scale synthesis and stability of COFs. In addition, imine-linked, rhombic Py-DHPh, Py-2,3-DHPh, Py-2,2′-BPyPh, and Py-3,3′-BPyPh COFs were synthesized by the reversible condensation reaction of *C*_2_ symmetric 4,4′,4″,4′″-(pyrene-1,3,6,8-tetrayl)tetraaniline bearing four amino groups at the vertices and *C*_2_-symmetric 2,5-dihydroxyterephthalaldehyde, 2,3-dihydroxyterephthalaldehyde, [2,2′-bipyridine]-5,5′-dicarbaldehyde, and [3,3′-bipyridine]-6,6′-dicarbaldehyde, respectively, at 120 °C for 3 days [129]. The BET surface areas as high as 1895 m^2^/g, 1932 m^2^/g, 2349 m^2^/g, and 2200 m^2^/g corresponded to Py-DHPh COF, Py-2,3-DHPh COF, Py-2,2′-BPyPh COF, and Py-3,3′-BPyPh COFs. Moreover, their substantially high porosity, thermal, and chemical stabilities permit the immobilization of metal ions, such as vanadium, palladium, and iridium, and create hybrid frameworks as heterogeneous catalysts for sulfide oxidation, Prins reaction, C–H functionalization, and Heck-epoxidation tandem reactions with excellent reusability and recyclability [130,131,132,133]. 

In addition to olefin-linked and imine-linked COFs, Dalapati et al. reported azine-linked rhombic Py-Azine COF by the reversible condensation of hydrazine and 1,3,6,8-tetrakis(4-formylphenyl)pyrene at 120 °C for 7 days [106]. The pyrene units were located at the vertices, whereas diazabutadiene linkers occupied the edges of the rhombic-shaped layer structure (Figure 6a). The framework was characterized by physiochemical analysis and displayed a BET surface area and pore size of 1210 m^2^/g and 1.76 nm, respectively.

### 4.2. Tetragonal COFs

Tetragonal COFs are usually constructed by stitching *C*_2_- and *C*_4_-symmetric building blocks. Dynamic imine, olefin, and boronate ester coupling reactions are widely employed to rationalize [*C*_4_ + *C*_2_] and [*C*_4_ + *C*_4_] topologies. For instance, Jiang and co-workers synthesized tetragonal metallophthalocyanine 2D MPc-COFs (M = Co, Cu, Ni, and Zn) via the boronate esterification reaction of 1,4-benzenediboronic acid and [OH]_8_PcM (M = Co, Cu, Ni, and Zn) under solvothermal conditions. CoPc-COF, CuPc-COF, NiPc-COF, and ZnPc-COF displayed disk-like morphologies and showed type-I reversible sorption isotherms, resulting in BET surface areas of 517 m^2^/g, 1360 m^2^/g, 624 m^2^/g, and 985 m^2^/g, respectively [134,135]. Remarkably, MPc-COFs prefer the slipped-AA stacking mode in contrast to the eclipsed AA-stacking manner found in most frameworks, thereby diminishing metal–over-metal electron conduction. This structural feature allows the conduction route of MPc-COFs, following the order CuPc-COF < ZnPc-COF < CoPc-COF. In addition, an electron-deficient 2D tetragonal boronate-ester-linked NiPc-BTDA COF was designed by connecting (2,3,9,10,16,17,23,24-octahydroxyphthalocyaninato)nickel(II) and 1,4-benzothiadiazole diboronic acid under solvothermal conditions. The NiPc-BTDA framework showed a BET surface area and pore width of 877 m^2^/g and 2.2 nm, respectively. Owing to the slipped AA-stacking mode and electron-deficient environment, the NiPc-BTDA COF displayed panchromatic photoconductivity with an electron mobility of 0.6 cm^2^V^−1^s^−1^, surpassing the similar 2D tetragonal boronate ester framework [136]. 

The profound roles of tuning pore size, pore environment, and structural and electronic features were further highlighted by Dichtel and co-workers. ZnPc-Py COF, ZnPc-DPB COF, ZnPc-NDI COF, and ZnPc-PPE COF were synthesized by the boronic ester linkage of zinc octahydroxyphthalocyanine with pyrene diboronic acid, diphenyl butadiynediboronic acid, napthalenediimide diboronic acid, and benzene-1,4-bis(phenylethynyl)diboronic acid, respectively, under solvothermal conditions [137]. The incorporation of an elongated diboronic acid building block expanded the diagonal pore widths of ZnPc COFs from 2.7 nm to 4.4 nm. The BET surface areas of ZnPc-Py COF, ZnPc-DPB COF, ZnPc-NDI COF, and ZnPc-PPE COF were 420 m^2^/g, 485 m^2^/g, 490 m^2^/g, and 440 m^2^/g respectively. Furthermore, ZnPc-Py COF and ZnPc-NDI COF crystallized as needle-like structures, whereas ZnPc-DPB COF and ZnPc-PPE COF formed rough irregular sheets and smooth aggregated spheroids. To bolster stability and biomimetic chemistry in a tetragonal framework, Jiang’s research group reported nonfunctionalized (MP-Ph COF, M = H_2_, Cu, and Ni) and functionalized (MP-DHPh*_x_* COF, M = H_2_, Cu, and Ni; *x* = 25%, 50%, 75%, and 100%) (Figure 6b) imine-linked porphyrin COFs. The successive installation of 2,5-dihydroxyterephthaldehyde in a MP-Ph COF (M = H_2_, Cu, and Ni) significantly influenced crystallinity through hydrogen-bonding interactions [138]. For example, CuP-Ph without hydrogen-bonding interactions displayed a poor crystallinity. On the contrary, CuP-DHPh COF with hydrogen-bonding interactions presented an excellent crystallinity. The hydrogen-bonding interaction in MP-DHPh*_x_* COFs (M = H_2_, Cu, and Ni; *x* = 25%, 50%, 75%, and 100%) prompted a planar conformation, which bestowed strong π-stacking interactions and significantly enhanced the extent of the crystallinity and porosity. For instance, the BET surface area exponentially increased from 83 m^2^/g (CuP-DMPh) to 365 m^2^/g, 485 m^2^/g, 762 m^2^/g, and 1094 m^2^/g with an increase in 2,5-dihydroxyterephthaldehyde content. In addition, Banerjee and co-workers reported the influence of intramolecular hydrogen-bonding interactions to enhance the crystallinity and chemical/thermal stability of tetragonal COFs [139,140]. Bifunctional tetragonal catechol-porphyrin COFs (2,3-DhaTph and 2,3-DmaTph) were synthesized by a reversible Schiff-base reaction of *C*_4_ symmetric 5,10,15,20-tetrakis(4-aminophenyl)-21H,23H-porphine with 2,3-dihydroxyterephthalaldehyde and 2,3-dimethoxyterephthalaldehyde, respectively. 2,3-DhaTph and 2,3-DmaTph COFs displayed excellent crystallinity, thermal stability, and chemical stability properties (3N HCl and 3N NaOH) due to the *trans*-conformation of imine bonds and intramolecular hydrogen-bonding interactions (O―H⋯N=C) [140]. Remarkably, the BET surface area of 2,3-DmaTph COF (668 m^2^/g, 1.4 nm) was lower compared to that of 2,3-DhaTph COF (1019 m^2^/g, 2.2 nm) due to the twisted conformation of phenyl rings. In addition, [*C*_4_ + *C*_2_] and [*C*_2_ + *C*_2_] topologies were imitated in tetragonal COF-366-M (M = H, Co), COF-367-M (M = Co, Co/Cu), and ILCOF-1, respectively [141,142,143]. Moreover, [*C*_4_ + *C*_4_] enabled the synthesis of tetragonal microporous porphyrin-*co*-phthalocyanine COF with a high density of π-columns. The polycondensation reaction of phthalocyanine with eight hydroxyl groups, M_2_Pc[OH]_8_ (M_2_ = Ni, Cu) and tetraphenyl porphyrin with four boronic acids, and M_1_TP_BA_P (M_1_ = H_2_, Zn, Cu) generated M_1_TTP-M_2_Pc COFs under solvothermal conditions [144]. The extended 2D sheet was crystallized via noncovalent interactions with unidirectional open nanochannels (1.8 nm). More importantly, the [*C*_4_ + *C*_4_] topology experienced a substantial decrease in the pore size as compared to the [*C*_2_ + *C*_2_] topology. In addition to a single-stage strategy, the double-stage strategy allowed the combination of boronate-ester- and imine-linked microporous CuPc-FPBA-ETTA COFs (1.8 nm) and mesoporous CuPc-FPBA-PyTTA (2.1 nm) frameworks and boronate-ester- and hydrazone-linked mesoporous CuPc-FPBA-DETHz COFs (3.7 nm) [145]. 

### 4.3. Hexagonal COFs

Hexagonal COFs were prepared by a reversible condensation reaction between organic building blocks with the precise positioning of binding units in a closed system. Dynamic imine-linked hexagonal pore frameworks with varying pore sizes and a tunable electronic and steric environment benefitted from the innumerable advantages of the bottom-up approach. The hexagonal COF-LZU1 was synthesized by the Schiff-base reaction of *C*_3_-symmetric 1,3,5-triformylbenzene and *C*_2_-symmetric 1,4-diaminobenzene under solvothermal conditions. The stable and porous nature (410 m^2^/g) of COF-LZU1 provoked post-synthetic modification by reacting with Pd(OAc)_2_. Pd/COF-LZU1 served as a heterogeneous catalyst for a Suzuki–Miyaura coupling reaction between phenylboronic acid and electron-donating or electron-withdrawing aryl halide [146]. In addition, Banerjee’s research group emphasized the significance of building block planarity to construct highly crystalline and porous hexagonal COFs. Hexagonal-pore 2,3-DhaTta was synthesized by the Schiff-base reaction of *C*_3_-symmetric 1,3,5-tris(4-aminophenyl)triazine with a planar core (triazine), and *C*_2_-symmetric 2,3-dihydroxyterephthalaldehyde exhibited a high crystallinity and porosity (1700 m^2^/g). In contrary, a hexagonal 2,3-DhaTab COF was synthesized by the condensation reaction of 1,3,5-tris(4-aminophenyl)benzene with a non-planar core (benzene), and 2,3-dihydroxyterephthalaldehyde displayed a low crystallinity and porosity (413 m^2^/g) [147]. This is attributed to the strong π–π stacking interaction between the hexagonal layers in the 2,3-DhaTta COF due to the three phenyl rings’ connection to the central triazine core (the torsion angles were 174.7°, 176.3°, and 179.9°) versus the non-planar core (the torsion angles were 145.5°, 149.6°, and 154.5°) in the 2,3-DhaTab framework. To demonstrate the fundamental role of interlayer interactions in COF stabilization, Jiang and co-workers reported the fabrication of a hexagonal TPB-DMTP COF by connecting *C*_3_-symmetric 1,3,5-tris(4-aminophenyl)benzene and *C*_2_-symmetric 2,5-dimethoxyterephthalaldehyde. The introduction of two electron-donating methoxy groups to the phenyl edge delocalized the lone pairs from oxygen over the phenyl ring and strengthened the interlayer interaction to stabilize the framework (the stacking energy of TPB-DMTP COF = 106.862 kcal/mol) [148]. However, a TPB-TP COF, constructed by using terephthalaldehyde instead of 2,5-dimethoxyterephthalaldehyde, possessed a low stacking energy of 94.084 kcal/mol. Furthermore, the BET surface area of the TPB-DMTP COF (2105 m^2^/g) was significantly higher than that of TPB-TP COF (610 m^2^/g).

To further strengthen the stability of hexagonal COFs, Banerjee’s research group synthesized a wide array of β-ketoenamine hexagonal COFs by the condensation reaction of *C*_3_-symmetric 1,3,5-triformylphloroglucinol and *C*_2_-symmetric amines. TpPa-1 and TpPa-2 COFs were prepared by the reversible Schiff-base reaction of 1,3,5-triformylphloroglucinol with 1,4-phenylenediamine and 2,5-dimethyl-1,4-phenylenediamine, respectively, followed by an irreversible enol-to-keto tautomerism [113]. Notably, TpPa-1 and TpPa-2 COFs exhibited good thermal stabilities and displayed an extraordinary resistance towards boiling water, acid, and base. The BET surface areas and CO_2_ uptake of TpPa-1 and TpPa-2 COFs were 535 m^2^/g and 78 cm^3^/g and 339 m^2^/g and 64 cm^3^/g, respectively. In addition, the judicious choice of either electron-withdrawing 2,3,5,6-tetrafluoro-1,4-phenylenediamine, 3,3′-dinitrobenzidine, 2,5-diaminobenzenesulfonic acid, and 2,5-diamino-1,4-disulfonic acid or functionalized 4,4′-azodianiline, 4,4′-diaminostilbene dihydrochloride, 2,2′-bipyridine-5,5′-diamine, and 2,6-diaminoanthraquinone building blocks created hexagonal TpPa-F_4_, TpBD-(NO_2_)_2_, NUS-9, NUS-10, Tp-Azo, Tp-Stb, TpBpy, and DAAQ-TFP COFs with tunable pore sizes and electronic properties [149,150,151,152,153]. Furthermore, a 2D hexagonal ionic EB-COF:Br COF was fabricated by the solvothermal reaction of 1,3,5-triformylphloroglucinol and ethidium bromide at 120 °C. In particular, the introduction of PW_12_O_40_^3−^ into the hexagonal pores of the cationic framework enhanced proton conductivity by 100 times compared to the as-synthesized EB-COF:Br at ambient temperature conditions [154]. In addition to imine and β-ketoenamine COFs, Yaghi and co-workers reported the creation of hydrazone-linked hexagonal COF-42 (Figure 6c) and COF-43 by the solvothermal reaction of 2,5-diethoxyterephthalohydrazide with 1,3,5-triformylbenzene and 1,3,5-tris(4-formylphenyl)benzene, respectively. COF-42 and COF-43 displayed good thermal stability properties (280 °C), and BET surface areas and pore sizes of 710 m^2^/g and 2.8 nm and 620 m^2^/g and 3.8 nm, respectively [105]. Furthermore, connecting the same hydrazide with 1,3,5-tris(4-formylphenyl)triazine under solvothermal conditions afforded a mesoporous hydrazone-linked hexagonal TFPT-COF (3.8 nm) [155]. JLU COF-4 was synthesized by the incorporation of *C*_3_-symmetric 1,3,5-triformylphloroglucinol and *C*_2_-symmetric 2,5-dimethoxyterephthalohydrazide, showing high crystallinity and porosity with a BET surface area of 923 m^2^/g [156]. The structural tunability and versatility highlighted azine-linked hexagonal COFs. Yu’s research group reported hexagonal pore, azine-linked ACOF-1 and COF-JLU2 synthesized by the solvothermal reaction of hydrazine hydrate with 1,3,5-triformylbenzene and 1,3,5-triformylphloroglucinol, respectively. Notably, ACOF-1 and COF-JLU2 displayed a high adsorption selectivity for CO_2_ over N_2_ and CH_4_ and BET surface areas of 1126 m^2^/g and 415 m^2^/g, respectively [107,157,158]. Furthermore, Liu and Lotsch’s research group underscored the pivotal role of hydrogen bonding and dihedral angle/planarity in the construction of highly porous and crystalline, azine-linked, hexagonal pore COF-JLU3 and N*_x_*-COFs (*x* = 0, 1, 2, 3) [159,160].

Yaghi and co-workers reported the first hexagonal pore frameworks, COF-1 and COF-5, synthesized by the self-condensation of 1,4-phenyldiboronic acid and the co-condensation of hexahydroxy triphenylene and 1,4-phenyldiboronic acid. COF-101 and COF-5 displayed high thermal stability (400 °C) and BET surface areas of 711 m^2^/g and 1590 m^2^/g, respectively [10]. The versatility of boronic-ester-linked hexagonal COFs was highlighted by the co-condensation reaction between 2,3,6,7,10,11-hexahydroxytriphenylene with 1,3,5-benzenetriboronic acid, 1,3,5-benzenetris(4-phenylboronic acid), and 4,4′-biphenyldiboronic acid, affording COF-6 (C_8_H_3_BO_2_), COF-8 (C_14_H_7_BO_2_), and COF-10 (C_6_H_3_BO), respectively. COF-6, COF-8, and COF-10 exhibited high thermal stability (450 °C) and BET surface areas of 980 m^2^/g, 1400 m^2^/g, and 2080 m^2^/g, respectively [125]. The hexagonal topology can be widely observed in stable imide-linked COFs [161]. For instance, Yan’s research group reported hexagonal polyimide crystalline COFs, PI-COF-1, PI-COF-2, and PI-COF-3, by the solvothermal reaction of *C*_2_-symmetric pyromellitic dianhydride and *C*_3_-symmetric tris(4-aminophenyl)amine, 1,3,5-tris(4-aminophenyl)benzene, and 1,3,5-tris [4-amino(1,1-biphenyl-4-yl)]benzene, respectively, at 200 °C or 250 °C for 5–7 days. PI-COFs showed high thermal stability over 500 °C and BET surface areas of 1027 m^2^/g and 1297 m^2^/g for PI-COF-1 and PI-COF-2, respectively, and 2346 m^2^/g for PI-COF-3 [109]. In addition to imine, imide, and β-ketoenamine COFs, the trimerization of symmetric aromatic cyanides stimulates triazine-linked frameworks. Kuhn et al. reported a hexagonal pore CTF-1 prepared by the trimerization of dicyanobenzene in the presence of ZnCl_2_ at 400 °C for multiple days. CTF-1 showed a higher thermal stability and a surface area of 791 m^2^/g [108]. Recently, Yaghi and co-workers reported the creation of an olefin-linked hexagonal COF-701 by Aldol condensation between 2,4,6-trimethyl-1,3,5-triazine and 4,4′-biphenyldicarbaldehyde under solvothermal conditions. COF-701 showed a remarkably high thermal stability (400 °C) and chemical stability in a base (KOH) and Brønsted acid [119]. More importantly, COF-701 displayed a large surface area and pore size of 1366 m^2^/g and 1.14 nm, respectively.

### 4.4. Trigonal COFs

Triangular frameworks constructed by the incorporation of *C*_6_-symmetric building blocks are rare in the field. For instance, Jiang and co-workers reported triangular HPB-COF (Figure 6e) and HBC-COF by the Schiff-base solvothermal reaction of terephthalaldehyde with *C*_6_-symmetric hexaphenylbenzene and hexabenzocoronene, respectively [162]. *C*_6_-symmetric vertices play a critical role in controlling interlayer interactions. HPB-COF and HBC-COF displayed excellent thermal stability (500 °C) and chemical stability in HCl, NaOH, and protic and aprotic solvents. The BET surface areas and pore sizes of HPB-COF and HBC-COF were 965 m^2^/g and 1.2 nm and 469 m^2^/g and 1.8 nm, respectively. In addition, the dual-pore triangular HAT-COF and HFPTP-BPDA COFs with pore sizes of 1.13 nm and 1.52 nm and 1.27 nm and 1.55 nm, respectively, were constructed via solvothermal reactions between *C*_3_-symmetric hexaazatriphenylene and 2,3,6,7,10,11-hexakis(4-formylphenyl)triphenylene and *C*_2_-symmetric terephthalaldehyde and 1,1′-biphenyl-4,4′-diamine, respectively [163,164]. Remarkably, the BET surface area of HFPTP-BPDA COF (1024 m^2^/g) is higher compared to that of HAT-COF (486 m^2^/g), possibly due to the incorporation of an elongated linker. In addition to imine, azine-linked HEX-COF 1 and sp^2^ carbon-linked CCP-HATN frameworks were designed by a reversible Schiff-base reaction and Knoevenagel condensation reaction, respectively [165].

### 4.5. Heteropore COFs

COFs with two or three different types of pores can be designed via the connection of organic building blocks of *D*_2h_, *C*_2_, and *C*_4_, with varying symmetricity and point groups [166,167] (Figure 6f). Furthermore, the topology of COFs is usually dictated by the symmetricity of the monomer units employed for the condensation reaction. Dual-pore frameworks with a Kagome lattice were constructed by the solvothermal reaction of *D_2h_*-symmetric, 4,4′,4′’,4′″-(ethene-1,1,2,2-tetrayl)tetraamine, and *C_2_*-symmetric terephthaldehyde in dioxane at elevated temperatures. These dual-pore frameworks displayed intense peaks in the PXRD profile and BET surface area of 1771 m^2^/g, implying good crystallinity and porosity. In addition, the frameworks displayed good thermal stability (400 °C) and chemical stability in common organic solvents [166]. In addition to dynamic imine chemistry, McGrier and co-workers illustrated the integration of rigid π-conjugated building units into highly ordered columnar periodic arrays. The solvothermal reaction of C_3_-symmetric dehydrobenzoannulenes, DBA_[12]_ and DBA_[18]_, with C_2_-symmetric 1,4-benzenediboronic acid in dioxane/mesitylene (2:1) at 105 °C for 3 days led to the creation of DBA COF-1 and DBA COF-2, respectively. The crystalline DBA COF-1 and DBA COF-2 displayed permanent porosity with BET surface areas and pore sizes of 1952 m^2^/g and 3.2 nm and 984 m^2^/g and 3.6 nm, respectively. Notably, the BET surface area of DBA COF-1 was larger than that of the well-known COF-5 (1590 m^2^/g), NTU-COF-2 (1619 m^2^/g), COF-10 (1760 m^2^/g), and TT-COF (1810 m^2^/g) [168]. Continuing with the same coupling chemistry, Zhang and co-workers illustrated a macrocycle-to-framework strategy using multitopic building blocks. The solvothermal reaction of 1,4-benzenediboronic acid with multitopic linkers of varying lengths, AEM-1 (9.3 Å) and AEM-2 (13.2 Å), in mesitylene/dioxane (1:1) at 100 °C for 7 days afforded AEM-COF-1 and AEM-COF-2, respectively. The highly crystalline frameworks were characterized using physiochemical analysis with BET surface areas and pore sizes of 1445 m^2^/g and 3.4 nm and 1487 m^2^/g and 3.9 nm, respectively, which is comparable to first-ever framework, COF-5 (1517 m^2^/g) [169]. Zhao’s research group underlined TP-COF-BZ to TP-COF-DAB transformation via in situ 1,4-diaminobenzene linker exchange using [1,1′:3′,1″-terphenyl]-3,3″,5,5″-tetracarbaldehyde, in which a benzene unit is chosen as the core and two aldehyde groups are introduced to the ends of its two branches. Furthermore, TP-COF-DAB and TP-COF-BZ displayed good thermal stability (>300 °C) and exhibited BET surface areas and pore sizes of 303 m^2^/g and 1.61 nm, 3.18 nm and 519 m^2^/g, and 2.56 nm and 3.91 nm, respectively [170]. The same research group further expanded the rational selection of building units to fabricate frameworks with three different types of pores using a heterostructural, mixed-linker strategy. The solvothermal reaction of 4,4′,4″,4′″-ethene(1,1,2,2-tetrayl)tetraaniline with terephthaldehyde and biphenyldialdehyde and biphenyldialdehyde and [1,1′:4’,1′’-terphenyl]-4,4″-dicarbaldehyde in varying stoichiometric rations afforded SIOC-COF-1 and SIOC-COF-2, respectively. Furthermore, chemically and thermally stable SIOC-COFs displayed surface areas and pore sizes in the range of ~475 m^2^/g and 7.3 Å, 11.8 Å, and 30.6 Å, which are fairly lower values than those shown by dual-pore COF-BPDA (447 m^2^/g) and COF-TPDA (84 m^2^/g) [171]. In another work, Lotsch and co-workers reported dual-pore PT_2_B-COF and PY_2_B-COF (pore size = 1.83 nm and 2.37 nm) by connecting *C*_2_-symmetric tetratopic rectangular linkers, benzidine linker, and *C*_3_-symmetric triangular tritopic linker in a 1:2:1 molar ratio. The BET surface areas and absolute CO_2_ uptake capacities of the dual-pore PT_2_B-COF and PY_2_B-COF are 2367 m^2^/g and 85 mgg^−1^ and 1984 m^2^/g and 127 mgg^−1^, respectively [172].

To expand the rational design of heteropore COFs via a de novo approach, Zhao’s research group designed dual-pore COF-BABD-DB and COF-BABD-BZ by the judicious choice of *C*_2v_-symmetric 4′-(bis(4-formylphenyl)amino)-[1,1′-biphenyl]-3,5-dicabaldehyde and *C*_2_-symmetric 1,4-diaminobenzene or benzidine. The crystalline COF-BABD-DB and COF-BABD-BZ displayed BET surface areas and pore sizes of 569 m^2^/g and 0.98 nm, 1.72 nm and 750 m^2^/g, and 1.8 nm and 2.5 nm, respectively. More importantly, the dual-pore COF-BABD-DB and COF-BABD-BZ exhibited high selectivity and sensitivity for the detection of 2,4,6-trinitrophenol over other nitro compounds with Stern–Volmer quenching constant (K_sv_) values of 5.7 × 10^5^ M^−1^ and 4.5 × 10^5^ M^−1^, respectively [173]. In another work, Ma and co-workers reported the comparison of dual-pore COF-ETTA-EDDA and BPyDC-ETTA COF and single-pore COF-PY-EDDA and Py-2,2′-BPyDC COFs as novel platforms for lipase immobilization and palladium docking for the kinetic resolution of secondary alcohol and C–H to C–X (X = Br, Cl, and I) and C–O functionalization with excellent reusability and recyclability [174,175]. Furthermore, the dual-pore (BPyDC)*_x%_*-ETTA COF (*x* = 0%, 25%, 50%, 75%, and 100%) with a controllable bipyridine content produced a palladium content, underlining the role of tricomponent condensation under solvothermal conditions. 

### 4.6. Three-Dimensional COFs

Three-dimensional COFs of different topologies were designed by connecting [*T*_d_ + *T*_d_], [*T*_d_ + *C*_3_], [*T*_d_ + *C*_4_], [*T*_d_ + *C*_2_], and [*C*_2_ + *C*_3_] building blocks. Yaghi and co-workers reported the first-ever boroxine- and boronic-ester-linked 3D COFs (Figure 6g) using solvothermal synthesis methodology. The self-assembly of tetrahedral (*T*_d_) tetra(4-dihydroxyborylphenyl)methane or tetra(4-dihydroxyborylphenyl) silane and by the condensation of *C*_3_-symmetric 2,3,6,7,10,11-hexahydroxytriphenylene favored the formation of 3D COF-102, COF-103, COF-105, and COF-108 by targeting two framework types (ctn and bor) based on triangular and tetrahedral nodes [176]. Furthermore, the 3D frameworks exhibited high thermal stability (400 °C to 500 °C), low density (0.17 g/cm^3^ for COF-108), and high porosity (COF-102 = 3472 m^2^/g and COF-103 = 4210 m^2^/g). Notably, the ctn structures were thermodynamically preferred, with 11.2 kcal/mol, over the bor topology due to a lower steric strain. In another work, McGrier’s research group reported boronate-ester-linked DBA-3D-COF1 (bor) by the solvothermal reaction of *T*_d_-symmetric tetra(4-dihydroxyborylphenyl)methane and *C*_3_-symmetric dehydrobenzoannulene at 95 °C for 3 days. The presence of a triangular-shaped π–electron-conjugated macrocycle underwent metalation in the presence of Ni(COD)_2_ at ambient temperature. Ni-DBA-3D-COF revealed a lower BET surface area (4763 m^2^/g) compared to DBA-3D-COF 1 (5083 m^2^/g), albeit both frameworks displayed high uptake capacities for ethane and ethylene [177]. To further explore the dynamic linkages and stability of 3D COFs, imine-linked COF-300 and COF-320 with a dia topology were conceived by connecting *T*_d_-symmetric tetra(4-anilyl)methane and *C*_2_-symmetric terephthalaldehyde under solvothermal conditions. COF-300 and COF-320 showed high thermal stability (490 °C) and permanent porosities of 1360 m^2^/g and 2400 m^2^/g, respectively [104,178]. The same dynamic linkage and topology with the uniform decoration of pyridyl functionalities was highlighted by Wang and co-workers. LZU-301 was synthesized through the reversible imine condensation of 3,3′-bipyridine-6,6′-dicarbaldehyde and tetra(4-anilyl)methane in the presence of a catalyst. The interpenetrated diamond network exhibited a remarkably high thermal stability (512 °C) and porosity (654 m^2^/g) [179]. The presence of pyridyl functionalities in LZU-301 showed a critical role in CO_2_ adsorption (1.59 mmol/g) at 298K and 1 bar and served as a versatile heterogeneous catalyst for the Knoevenagel condensation between aromatic aldehyde and malononitrile. The broadly reported dia network was further described in 3D ionic COFs. Three-dimensional ionic COF-1 and three-dimensional ionic COF-2 were synthesized by the Schiff-base reaction of *T*_d_-symmetric tetrakis(4-formyl)methane with *C*_2_-symmetric diimidium bromide and ethidium bromide, respectively [180]. Three-dimensional ionic frameworks exhibited rod-like morphology and high thermal stability (450 °C). Furthermore, the BET surface areas and CO_2_ adsorption values for 3D ionic COF-1 and 3D ionic COF-2 were 966 m^2^/g and 93 mg/g and 880 m^2^/g and 76 mg/g, respectively.

In addition to imine-, boroxine-, and boronate-ester-linked COFs, 3D frameworks with dia topologies designed by using imide linkage have a pivotal role in the research [161]. PI-COF-4 and PI-COF-5 (Figure 6h) were synthesized by the solvothermal reaction of pyromellitic dianhydride with 1,3,5,7-tetraaminoadamantane and tetrakis(4-aminophenyl)methane, respectively [181]. Furthermore, 3D chiral (*R*,*R*)-CCOF 5, (*R*,*R*)-CCOF 6, and salphen-based JUC-509-Y (Y = Mn, Cu, or Eu) synthesis elucidated the structural diversity and pore tunability of 3D COFs. Notably, 3D PI-COF-4 and PI-COF-5 served as ibuprofen, captopril, and caffeine drug carrier, whereas JUC-509-Cu displayed a high performance in the removal of superoxide radicals without any hinderance in recyclability and reusability [182,183]. This underscored the fundamental role of the building blocks’ expansion to fabricate crystalline, porous, and thermally stable 3D COFs to address the challenges in the fields of biology and medicine. In addition to a dia network, Wang and co-workers reported an imine-linked 3D-Py-COF with a pts topology. The [4 + 4] imine condensation reaction was conducted between *C*_2_-symmetric 1,3,6,8-tetrakis(4-formylphenyl)pyrene and *T*_d_-symmetric tetra(*p*-aminophenyl)methane under solvothermal conditions at 120 °C for 3 days. Three-dimensional-Py-COF with a granular morphology exhibited high thermal stability (550 °C), porosity (1290 m^2^/g), and selective adsorption of CO_2_ over N_2_. More importantly, 3D-Py-COF showed the chemosensing of picric acid with a Stern–Volmer quenching constant *K*_sv_ of 3.1 × 10^4^ M^−1^ [184]. In another work, Wang’s research group synthesized 3D-Por-COF (M = H_2_, Cu) and 3D-TPE-COF with a pts network by the dynamic imine condensation reaction of tetra(*p*-aminophenyl)methane with 5,10,15,20-tetrakis(4-benzaldehyde)porphyrin and 1,1,2,2-tetrakis(4-formyl-(1,1′-biphenyl))ethene, respectively, under solvothermal conditions for 7 days. The thermal (400 °C) and chemical stability of 3D-Por-COF, 3D-CuPor-COF, and 3D-TPE-COF in conjunction with a high surface area of 1398 m^2^/g, 1335 m^2^/g, and 1084 m^2^/g, respectively, highlighted the advancement in formulating novel topologies and synthetic techniques [185,186]. In addition to the widely described topologies in 3D COFs, srs anionic silicate 3D COF and ctn β-ketoenamine 3D BF-COF-2 are sporadic networks obtained from porous framework chemistry, thereby highlighting the importance of the judicious synthesis of symmetric building blocks and COFs via a de novo approach [187,188].

The stitching of rationally selected, rectangular-planar, and trigonal-planar building blocks with conformational strains creates crystalline 3D COFs with novel topologies. Cui and co-workers reported the twofold interpenetrated ffc topology COF-1 and COF-2 by a [3 + 4] condensation reaction between triangular 4′,4′″,4′″″-Nitrilotris(([1,1′-biphenyl]-4-carbaldehyde)) with rectangular 4,4′,4″,4′″-(Ethene-1,1,2,2-tetrayl)tetraaniline and triangular tris(4-aminophenyl)amine with rectangular 4,4′″,4′″″-(Ethene-1,1,2,2-tetrayl)tetrakis(([1,1′-biphenyl]-4-carbaldehyde)), respectively. Both COFs displayed excellent thermal stability (350 °C) and chemical stability in DMF, MeOH, HCl (aq), and NaOH (aq). Notably, COFs serve as efficient photocatalysts for the cross-dehydrogenative coupling reaction and the asymmetric α-alkylation of aldehydes integrated with a MacMillan imidazolidinone as the chiral catalyst under visible light [189]. Recently, Zhu et al. presented the construction of 3D COFs RICE 3-7 with pto and mhq-z topologies designed by triangular and rectangular building blocks, respectively. For instance, the Schiff-base reaction of triangular 1,3,5-tris(4-aminophenyl)benzene and rectangular 4,4′″,4′″″,4′″″″-(Ethene-1,1,2,2-tetrayl)tetrakis(([1,1′-biphenyl]-4-carbaldehyde created RICE-3 with pore size distributions of 1.0 nm, 1.4 nm, 3.2 nm, and 4.6 nm. Furthermore, COF RICE 3-7 displayed good carbon dioxide uptake, with RICE-5 exhibiting the highest adsorption capacity of 50cm^3^/g at 273K and 1 bar [190]. In addition to rectangular and triangular building blocks, the [4 + 4] condensation reaction of tetrahedral and quadrilateral monomer units affords a self-penetrated ljh (Luojia Hill) topology 3D-TPB-COF-Ph and a five-fold interpenetrated pts 3D-TPB-COF-OMe structure. The condensation reaction of tetra(*p*-aminophenyl)methane and 1,2,4,5-tetrakis(4-formylphenyl)-3,6-diphenylbenzene led to a bulky ljj structure 3D-TPB-COF-Ph through three four-connected nodes with a point symbol of {4⸳8^5^}_2_ {4^2^⸳8^4^}{8^6^}. Furthermore, 3D-TPB-COF-Ph exhibited excellent thermal (450 °C) and chemical stability and displayed a BET surface area of 1430 m^2^/g and a pore size of 1.0 nm. Notably, this example showed the tuning of 3D COF topology through steric control in the building blocks, which underlines the important role of structural diversity in the exploration of novel COF topologies [191].

The control alignment of building blocks via supramolecular assembly or predesigned rigid linkages is fundamental to comprehend the desired topology. Zhang et al. reported the design of eight-connected building blocks with cubic configuration, 5,10,15,20-tetrayl(tetrakis(([1,1′:3′,1″-terphenyl]-4,4″-dicarbaldehyde)))-porphyrin to reticulated into interpenetrated pcb-topology NUST-5 and NUST-6 by traditional Schiff-base condensation with *p*-phenylenediamine and dimethyl-*p*-phenylenediamine at elevated temperatures. Both COFs showed a uniform rod-like morphology with excellent thermal stability (400 °C). Furthermore, NUST-5 and NUST-6 serve as an effective photocatalyst for CO_2_ reduction with CO and CH_4_ production values of 54.7, 76.2 µmolg^−1^ and 17.2, 12.8 µmolg^−1^, respectively [192]. This strategy exemplified fundamental role of higher connective and symmetric building units to boost the structural advancement of 3D COFs. In addition to an eight-connected monomer unit, the utilization of numerous organic building blocks offers ample possibilities in the exploration of COFs with a novel topology. A three-dimensional-ceq-COF with a 6,3-connected ceq topology was constructed through the combination of a triangular linker and stereo-triangular prism vertex. The solvothermal reaction of 2,3,6,7,14,15-hexakis(4-formylphenyl)triptycene and 1,3,5-tris(4-aminophenyl)triazine in mesitylene/dioxane affords a 3D-ceq-COF with extraordinary thermal (550 °C) and chemical stability in protic and aprotic solvents. Notably, a two-fold interpenetrated 3D-ceq-COF featured a moderate surface area (1148 m^2^/g), microporosity (10 Å and 16 Å), and CO_2_ (91.27 cm^3^/g), CH_4_ (36.28 cm^3^/g), and H_2_ (178.49 cm^3^/g) uptake capacities [193]. Using the same stereo-triangular prism node, 2,3,6,7,14,15-hexakis(4-formylphenyl)triptycene, Fang and co-workers reported the construction of a non-interpenetrated acs-topology JUC-569 by connecting it with 2,3,6,7,14,15-hexa(3′,5′-diisopropyl-4′-amino)triptycene. This is due to the six-connected node and 60° link angle, which tends to form a six-connected non-interpenetrated acs topology. The JUC-569 exhibited a BET surface area of 1254 m^2^/g with a microscopic cavity of 1.87 nm. Moreover, the JUC-569 displayed moderate CO_2_ (47 cm^3^/g), CH_4_ (19 cm^3^/g), and H_2_ (274 cm^3^/g) uptake capacities [194]. This in-depth study showed the pivotal role of organic monomer units in the fabrication of 3D COFs with various topologies, thereby expanding the potential of frameworks in the greenhouse gas capture and energy storage. The diversity of frameworks topology was further shown by Fang and co-workers by synthesizing the first-ever triptycene-based 3D JUC-564 framework with an stp topology. The JUC-564 was constructed by connecting six-connected 2,3,6,7,14,15-hexakis(4′-formylphenyl)triptycene with a link angle of 60° and four-connected 1,3,6,8-tetrakis(4-aminophenyl)pyrene with a link angle of 120° at elevated temperatures. The interconnected channel system of JUC-564 displayed a record-breaking low density (0.118 gcm^−3^) and a surface area and pore size of 3383 m^2^/g and 43 Å, respectively. Due to the presence of ultra-large channels in the stp net framework, the JUC-564 exhibited an uptake capability of myoglobin (6.1 μmolg^−1^) with a dimension of 21 Å × 35 Å × 44 Å [195]. Continuing with the diversity of frameworks topology, the first-ever example of 3D COFs with an isoreticular tbo topology of well-known metal–organic frameworks, HKUST-1, was reported by Cui and co-workers. The dynamic imine condensation reaction of three-connected triangular 4′,4‴,4′″″-nitrilotris[(1,1′-biphenyl)-4-carbaldehyde] with four-connected square 5,10,15,20-tetra(4-aminophenyl)porphyrin and 5,10,15,20-tetrakis[(4-aminophenyl)porphinato]copper (II) created tbo network COF-1 and COF-2, respectively. The tbo-COFs consisted of three types of polyhedral cages: the first truncated tetrahedron (14 Å) was formed from four aldehyde monomers and twelve amine units; the second truncated cube (20 Å) was shaped from eight aldehyde and twenty-four amine units; and the largest cage, truncated cuboctahedron (33 Å) was formed from twenty-four aldehyde units. Both COFs showed good thermal stability (360 °C) and chemical stability in common organic solvents, including DMSO, THF, and MeOH. Remarkably, the frameworks serve as efficient, recyclable, and heterogeneous photocatalysts for the hydroxylation of arylboronic acids to phenols (over 90% yield) and the defluoroalkylation of trifluoromethyl aromatics with alkenes (over 80% yield) [196]. Recently, Xu et al. reported an unprecedented she topology constructed from D*_3d_*- and D*_4h_*-symmetric building blocks. The solvothermal reaction of D*_3d_*-symmetric hexa(4-formylphenyl)benzene and D*_4h_*-symmetric metal 5,10,15,20-tetra(4-aminophenyl)porphyrin (M = Co^2+^, Cu^2+^, and Fe^3+^) in the presence of an acid catalyst at elevated temperatures afforded an M-TAPP-HFPB-COF. Three-dimensional frameworks were characterized using various analytic approaches and displayed good thermal stability and chemical stability in water, hexane, methanol, dichloromethane, DMF, HCl (3M), and NaOH (3M). Furthermore, the BET surface areas of TAPP-HFPB-COF, Co(II)- TAPP-HFPB-COF, Cu(II)- TAPP-HFPB-COF, and Fe(III)- TAPP-HFPB-COF were 1060 m^2^/g, 865 m^2^/g, 826 m^2^/g, and 923 m^2^/g, respectively, with a uniform pore distribution of 20 Å. Notably, the 3D frameworks displayed effective catalytic activity for the substrate-specific light-induced α-functionalization of aldehydes for a range of aldehydes in low-to-moderate yield and a photocatalytic CO_2_ reduction with CO production of 140 mmolg^−1^ [197]. 

The photocatalytic efficacy of 3D COFs for oxidative amine coupling and cycloaddition reactions were examined by a novel **scu** topology NKCOF-25-X (X = H or Ni) [198]. The [8 + 4] construction approach using octatopic 4′,5′-bis(3,5-diformylphenyl)-3′,6′-dimethyl-[1,1′:2′,1″-terphenyl]-3,3″,5,5″-tetracarbaldehyde with eight aldehyde groups and tetratopic tetra(4-aminophenyl)porphyrin with four amino groups afforded NKCOF-25 at elevated temperatures. The same research group used a similar bottom-up strategy with the same octatopic aldehyde monomer unit, which afforded NKCOF 21-23 with an eight-connected **bcu** topology. The condensation reaction of 4′,5′-bis(3,5-diformylphenyl)-3′,6′-dimethyl-[1,1′:2′,1″-terphenyl]-3,3″,5,5″-tetracarbaldehyde with 1,4-phenylenediamine, benzidine, and 4,4′-diaminoterphenyl afforded NKCOF-21, NKCOF-22, and NKCOF-23, respectively. The NKCOF 21-23 displayed uniformly distributed block crystal and square pores with extremely high thermal stability. The BET surface areas and pore volumes were 1397 m^2^/g and 0.23 m^3^g^−1^, 1580 m^2^/g and 0.34 m^3^g^−1^, and 1900 m^2^/g and 1.05 m^3^g^−1^ for NKCOF-21, NKCOF-22, and NKCOF-23, respectively. Due to hydrogen-bond interactions (C—H∙∙∙∙N), NKCOFs displayed high ethane and ethylene adsorption at 273K, 298K, and 308K. For instance, NKCOF-21, -22, and -23 for ethene at 298K and 1 bar were 74.3 cm^3^/g, 40.7 cm^3^/g, and 51.0 cm^3^/g, respectively, while the adsorption capacity for ethane reached 97.9 cm^3^/g, 65.9 cm^3^/g, and 60.5 cm^3^/g, respectively. More importantly, bcu-network COFs displayed ethylene/ethane separation for 10 cycles with a complete retention of their structural robustness and crystallinity [199]. One of the fundamental challenges in the field of 3D COFs is the fabrication of frameworks with a specific topology and the rejection of other possibilities. The COF research community has developed synthetic strategies to construct organic building blocks with sufficient structural, symmetrical, and reactive information to target specific topologies. This was illustrated by Nguyen et al. by the selective construction of COF-790 with an fjh topology using triangle and square building blocks. The solvothermal reaction of triangular 1,3,5-trimethyl-2,4,6-tris(4-formylphenyl)benzene with square 1,1,2,2,-tetrakis(4-aminophenyl)ethene in nitrobenzene and mesitylene at 85 °C for 3 days afforded a crystalline 3D COF-790. Furthermore, the dihedral angle between organic monomer units was in the range of 75°–90°. For instance, the dihedral angle of aldehyde monomer due to the presence of methyl groups was at 74°, 83°, and 90°, thereby bolstering a framework with the desired topology. Notably, the stitching of aldehyde monomers without methyl group and with amine afforded an amorphous solid that has a paramount role in conformation processes. However, the same monomer under solvothermal conditions in an alternative reaction condition (1,2-dichlorobenzne, 1-butanol, and acetic acid, at 120 °C, for 3 days) afforded the tth topology COF-340. To further show the fundamental role of building block conformation, isoreticular COF-791 and COF-792 were constructed by connecting 1,3,5-trimethyl-2,4,6-tris(4-formylphenyl)benzene with 1,2,4,5-tetrakis(4-aminophenyl)benzene and 1,2,4,5-tetrakis(4-aminophenyl)-3′,6′-dimethylbenzne, respectively [200]. This bolstered the role of building blocks’ properties, reaction conditions, catalyst, and other parameters to construct COFs with numerous topologies. For instance, Cooper and co-workers reported nbo topology spiroborate-linked SPB-COF-DBA by the control alignment of building blocks. The solvothermal reaction of square–planar cobalt (II)phthalocyanine and trimethylborate in N,N-dibutylformamide at 120 °C for 3 days afforded a crystalline ionic SPB-COF-DBA. The cubic pores of the moderately stable SPB-COF-DBA displayed a BET surface area of 1726 m^2^/g [201].

## 5. Synthetic Methods

The judicious choice of building blocks and the directional nature of dynamic linkages are essential to construct crystalline and porous frameworks. A de novo approach creates the formation of robust, reversible covalent linkage, thereby bolstering the thermodynamic control of condensation reactions. However, a kinetically controlled reaction enables the irreversible bond. This self-healing procedure encompasses the error checking and proof reading of structures to afford stable networks without defects. In addition to the fundamental role of reversibility, irreversible coupling reactions, such as dioxin, olefin, and nucleophilic aromatic substitution, have been reported in the design of porous networks and expansion of the library of COFs via different synthetic routes [100,101,102,103].

### 5.1. Solvothermal Synthesis

**Figure 7:**
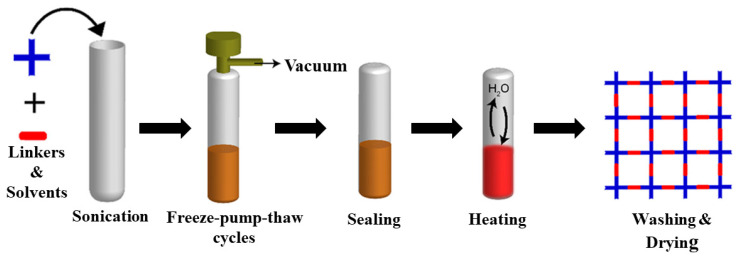
COF solvothermal synthetic methodology. Reprinted with permission from [41] (Copyright © American Chemical Society).

Solvothermal synthesis is one of the most widely used techniques for the synthesis of porous frameworks. In a typical protocol, organic monomers, solvents, and catalysts are placed in a Pyrex tube followed by sonication, degassed through freeze–pump–thaw cycles (liquid N_2_ bath), sealed using a burner, and set aside for the required amount of time at a suitable temperature. Then, the precipitate is collected, washed, and dried to produce fluffy, solid-powdered COFs (Figure 7). This method was the first and is the most commonly used procedure to synthesize two 2D boroxine-linked and boronate-ester-linked COFs. Moreover, this methodology was used to synthesize imine-linked COFs, hydrazone-linked COFs, and azine-linked COFs, as highlighted above. The degree of crystallization and porosity depend highly on the reaction time, solvent, catalyst, symmetricity/reactivity, and solubility of the building blocks. The thermodynamics of COF synthesis using the solvothermal method can provide enough energy to overcome the Gibbs free energy of crystallization. A prolonged reaction time and tedious stepwise synthetic route hinder the large-scale synthesis of frameworks; however, the growth of alternative methods is still in its infancy. For instance, TPT-COF-1 was prepared using a gram scale by the solvothermal reaction of 2,4,6-tris(4-aminophenoxy)-1,3,5-triazine and 2,4,6-tris(4-formylphenoxy)-1,3,5-triazine (TPT-CHO) with a BET surface area of 1589 m^2^/g [202]. 

### 5.2. Microwave Synthesis

The extended reaction time and synthetic conditions during solvothermal synthesis deter the large-scale synthesis of frameworks. An early approach to overcome this challenge was reported by Campbell et al. using microwave irradiation for COF fabrication. The rapid preparation (20 min) of COF-5 followed by purification and activation yielded a surface area over 2000 m^2^/g, which is higher than the surface value projected by the solvothermal synthesis [203]. The general method of preparing COFs via microwave synthesis initiated by mixing building blocks in a suitable solvent system under inert atmosphere in a sealed microwave tube. It is important to activate COFs with low-surface-tension solvents by the Soxhlet extraction to remove building blocks and monomers adsorbed in the pores. In addition to COF-5, azine-linked, β-ketoenamine-linked, and imide-linked COFs were synthesized using microwave irradiation [204,205,206]. For instance, Wei and co-workers reported the synthesis of β-ketoenamine TpPa-1 and TpPa-2 COFs under microwave irradiation within one hour. Notably, both COFs exhibited better crystallinity and porosity in comparison to the preparation via the solvothermal route. In another work, Lee et al. [206] conducted microwave-assisted PI-COF synthesis using pyromellitic dianhydride and tris(4-aminophenyl)amine.

### 5.3. Mechanochemical Synthesis

To take COF chemistry to an interdisciplinary level, it is essential to explore simple synthetic methods, thus avoiding any complicated conditions, such as a reaction in a sealed Pyrex tube, inert atmosphere, and elevated temperatures. A mechanochemical synthesis prompted the construction of COFs via stable covalent bonds through a simple, economical, and environment friendly route [207]. Despite the advantage of this synthetic route, the poor crystallization and amorphous nature of the as-synthesized COFs is a prime challenge; however, by the addition of either solvent or molecule organizer, such as *p*-toluenesulfonic acid frameworks, the crystallinity and porosity can be considerably improved. In the mechanochemical synthesis of β-ketoenamine TpPa-1, TpPa-2, TpPa-NO_2_, TpPa-F_4_, TpBD-(NO_2_)_2_, TpBD-(OMe)_2_, TpBpy, and other COFs, the building blocks were mechanically grinded using a mortar and pestle followed by heating at 170 °C for 1 min [153,208,209]. The yielded COFs exhibited less crystallinity and porosity compared to the frameworks synthesized using the solvothermal route. Moreover, sulfonic-acid-decorated NUS-9 and NUS-10 were constructed mechanochemically by the condensation reaction of 1,3,5-triformylphloroglucinol with 2,5-diaminobenzenesulfonic acid and 2,5-diaminobenzene-1,4-disulfonic acid, respectively, exhibiting low porosity and crystallinity [149]. 

### 5.4. Sonochemical Synthesis

The sonochemical method provides an alternative approach to the fabrication of frameworks with uncomplicated reaction conditions. The sonochemical method uses the process of cavitation, in which the reaction conditions, temperature, and pressure can be raised to initiate and accelerate the extent of polymerization. Yang and co-workers reported the use of the sonochemical method for COF-1 and COF-5 syntheses in a short reaction time (30 min–2 h) with high crystallinity and porosity (BET surface area = 2122 m^2^/g) [210]. Although this method demonstrates the boundless potential of constructing framework with high crystallinity and stability under mild conditions, it is not apt to study different dynamic linkages to expand the library of COFs. 

### 5.5. Ionothermal Synthesis

Covalent triazine frameworks (CTFs) synthesized using the ionothermal route are crystalline in nature; however, most CTFs are amorphous materials with the absence of a long-range order. In a usual method, organic building blocks of zinc chloride are placed in an ampule, which is sealed and heated to an elevated temperature (350–450 °C) for a long time (2–3 days). The solid product is washed with water and stirred in diluted hydrochloric acid to remove the unreacted monomers and zinc chloride [109]. Using this protocol, crystalline CTF-1 and CTF-2 were synthesized using cyanuric chloride via the trimerization reaction [211]. Recently, a milder and greener synthetic route was reported using ionic liquid as the solvent. For instance, 3D ionic-liquid-containing COFs (3D-IL-COF-1, 3D-IL-COF-2, and 3D-COF-3) were synthesized using 1-butyl-3-methylimidazolium bis-((trifluoromethyl)sulfonyl) imide as a green solvent to facilitate the rate of reaction between tetrakis(4-formylphenyl)methane with *p*-phenylenediamine, 4,4′-diaminobuiphenyl, and 4,4″-diamino-p-phenylenediamine, respectively [212]. 

### 5.6. Room-Temperature Synthesis

Most widely used COF synthetic methods require elevated temperatures and prolonged reaction times. The innumerable building blocks are unstable at high temperatures, and synthetic approaches can be hazardous and dangerous. In comparison to the solvothermal route, the room-temperature method offers simplicity and ease of operation. It is of profound importance to explore novel synthetic conditions, catalysts, and linkages to enable the large-scale synthesis of COFs. Within this context, the solution–suspension approach is employed to prepare COFs at room temperature. In this approach, 1,4-phenylenediamine and 1,3,5-triformylbenzene were dissolved in dioxane at room temperature, followed by the addition of acetic acid as the catalyst. The mixture was kept for 3 days at ambient temperature conditions to construct COFs with good crystallinity and porosity (410–1537 m^2^/g) [213]. This method is fast, effective, and can be used for the large-scale commercial production of frameworks for practical applications. These stable building blocks offer strong π interactions and high solubility to form COFs. The continuous flow of COF-LZU1 synthesis demonstrated a production rate of 41 mgh^−1^ at an extremely high space–time yield of 703 Kgm^−3^day^−1^. In another work, Bein and co-workers reported the synthesis of boroxine-based COF films from (4,6-diethoxybenzo[1,2-b:4,5-b′]dithiophene-2,6-diyl)diboronic acid and hexahydroxy triphenylene through the vapor-assisted conversion method. This method is based on the conversion of precursors in a cast solution layer into a continuous crystalline and porous film by exposure to a vapor of specific composition at moderate temperatures [214]. The role of time and vapor mixture is crucial for the formation of a highly regular structure. 

In another work, COF nanobars were synthesized in a carbondioxide (CO_2_)/water solvent. Compressed CO_2_ is cheap and nonflammable and can affect the properties of solvents to influence the structure and morphology of COFs. The first-ever report used the gram-scale method for preparing COF nanosheets with *n*-butyl lithium as the intercalation agent. Guan et al. reported imine-linked 3D interpenetrated dia structures, 3D-IL-COF-1, 3D-IL-COF-2, and 3D-COF-3, by suspending tetrakis(4-formylphenyl)methane with *p*-phenylenediamine, 4,4′-diaminobuiphenyl, and 4,4″-diamino-p-phenylenediamine, respectively, at ambient temperature in a 1-butyl-3-methylimidazolium bis((trifluoromethyl)sulfonyl)imide solvent [212]. Three-dimensional-IL-COFs displayed high crystallinity and stability, and a BET surface area of 517 m^2^/g for 3D-IL-COF-1, 653 m^2^/g for 3D-IL-COF-2, and 870 m^2^/g for 3D-IL-COF-3. This highlights the potential pathways of using ionic liquid as a green solvent for the large-scale synthesis of COFs. In addition to green solvent, Lewis acids, such as metal triflates, are effective in escalating the construction of imine-linked frameworks in ambient temperature conditions. Matsumoto et al. reported the room-temperature synthesis of imine-linked TAPB-PDA COF using Sc(OTf)_3_ (0.02 equivalents) in 20 min. In addition to Sc(OTf)_3_, other triflates, such as Eu(OTf)_3_, In(OTf)_3,_ Yb(OTf)_3_, Y(OTf)_3_, and Zn(OTf)_3_, in different ratios were tested and found ineffective to catalyzed crystalline TAPB-PDA frameworks [215]. In comparison to Sc(OTf)_3_, a conventional catalyst, such as acetic-acid-assisted solvothermal synthesis, requires elevated temperatures (70 °C) and long time (72 h), exhibiting a moderate degree of crystallinity and porosity (692 m^2^/g). In another work, Verduzco and co-workers reported the fabrication of imine-linked framework using transition-metal nitrate as the catalyst at room temperature. Six different catalysts, Fe(NO_3_)_3_⸱9H_2_O, Ni(NO_3_)_2_⸱6H_2_O, Zn(NO_3_)_2_⸱6H_2_O, Mn(NO_3_)_2_⸱6H_2_O, Cu(NO_3_)_2_⸱6H_2_O, and Co(NO_3_)_2_⸱6H_2_O, in varying stoichiometric ratios (1 mol%, 3 mol%, 5 mol%, 7 mol%, and 10 mol%) tried to synthesize TAPB-OMePDA COF through the condensation reaction of 1,3,5-tris(4-aminophenyl)benzene and 2,5-dimethoxyterephthalaldehyde at room temperature. Notably, 10 mol% of Fe(NO_3_)_3_⸱9H_2_O was found effective to synthesize the framework with the highest degree of crystallinity and porosity (1345 m^2^/g). In addition to Fe(NO_3_)_3_⸱9H_2_O, other nitrate salts were found either moderately or poorly effective to prepare crystalline framework. In addition to TAPB-OMePDA COF, TAPB-PDA, TAPB-BPDA, COF-V, TAPB-C8PDA, TAPT-PDA, and TAPT-OMePDA COFs successfully synthesized at ambient temperature with a high porosity after activation using supercritical CO_2_ [216]. Moreover, Zamora and co-workers highlighted the room temperature synthesis of crystalline yellow gels by connecting 1,3,5-tris(4-aminophenyl)benzene and benzene-1,3,5-tricarbaldehyde using acetic acid as a catalyst in either *m*-cresol or dimethyl sulfoxide (DMSO) [217]. 

**Figure 8:**
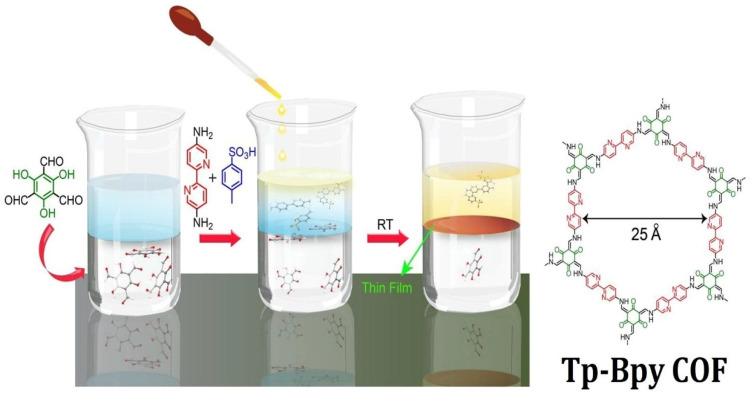
Synthesis of Tp-Bpy COF film via interfacial crystallization at room temperature. Reprinted with permission from ref. [218] (Copyright © American Chemical Society).

COF membrane synthesis was viable a decade after the first ever COF fabrication performed under a solvothermal condition. To date, the development of COF membranes is still in its early stages. The prominent drawbacks come from three aspects, including low crystallinity, inferior processability, and irregular pore channel size. As such, the interfacial polymerization of β-ketoenamine-linked COF membranes at room temperature has advanced as a prominent method to prepare COF membranes. Banerjee and co-workers fabricated Tp-Bpy, Tp-Azo, Tp-Ttba, and Tp-Tta COF membranes by the liquid–liquid interfacial polymerization of 1,3,5-triformylphloroglucinol with 2,2′-bipyridine-5,5′-diamine, 4,4′-azodianiline, 4,4′,4″-(1,3,5-triazine-2,4,6-triyl)tris(1,1′-biphenyl)trianiline, and 4,4′,4″-(1,3,5-triazine-2,4,6-triyl)trianiline, respectively, at ambient temperature [218]. In this method, 1,3,5-triformylphloroglucinol suspended in dichloromethane and amine monomer dissolved in water was employed as the bottom layer. Since dichloromethane and water are immiscible with each other, a liquid–liquid interface was generated at the interface to afford membranes on the top of the aqueous layer after 72 h (Figure 8). Tp-Bpy, Tp-Azo, Tp-Ttba, and Tp-Tta COFs displayed BET surface areas of 1151 m^2^/g, 647 m^2^/g, 626 m^2^/g, and 333 m^2^/g, respectively. Notably, porous and crystalline Tp-Bpy thin films exhibited an unprecedented acetonitrile permeance of 339 Lm^−2^h^−1^bar^−1^. The interfacial polymerization of a COF membrane is usually conducted in ambient conditions. In general, high temperature can increase the solubility of monomer units, but interrupts the interface morphology, which can lead to defects in the framework membrane. In addition to interfacial polymerizations at the liquid/liquid interface, interfacial polymerization at the liquid/air interface was established for the fabrication of COF membranes. Lai and co-workers reported the fabrication of a TFP-DHF 2D COF membrane from 1,3,5-triformylphloroglucinol and 9,9-dihexylfluorene-2,7-diamine through the Langmuir–Blodgett method [219]. A monomer layer was formed on the surface of water by spreading a toluene solution of amine and aldehyde monomer on the water, followed by the evaporation of toluene. The polymerization at the interface was catalyzed by trifluoroacetic acid into the water to form 3 nm of a TFP-DHF membrane at room temperature.

The challenges associated with the liquid–liquid interface encouraged the development of the present synthetic method. The in situ growth of COF membranes on a porous substrate or free-standing COF membrane offers a high separation performance. Jiang and co-workers reported a polydopamine-modulated in situ crystallization route to prepare sulfonated imine-linked TFP-DABA COF membranes on a polyacrylonitrile (PAN) substrate [220]. The polydopamine layer (10 nm) deposited on PAN contained numerous functional groups serving as linking functionalities that adsorbed and bound framework units to promote nucleation. The sequential addition of 1,3,5-triformylphloroglucinol and 2,5-diaminobenzenesulfonic acids in 1,4-dioxane and a water solution to the modified PAN produced a β-ketoenamine membrane after 72 h at room temperature. Notably, the SCOF/PDA/PAN membrane exhibited water permeance of up to 1346 Lm^−2^h^−1^MPa^−1^ with desirable dye rejection. Furthermore, a heterostructure COF bilayer membrane was fabricated by a mixed-assembly strategy under an ambient condition. The Jiang research group illustrated the assembly of a TpDHBD nanosheet on a polydopamine-modified PAN substrate by vacuum filtration. Subsequently, the TpHZ COF layer displayed an in situ growth on the framework nanosheet layer via the vapor–liquid interfacial synthesis method. This exclusive promotion of heterogeneous nucleation assisted the creation of a second TpHZ COF layer. Remarkably, the crystallinity of the bilayer membrane was greater as compared to the membrane fabricated by liquid–liquid interfacial polymerization due to the epitaxial growth of a COF nanosheet and slow diffusion of monomers. Furthermore, the heterostructural membrane displayed a separation factor of 4464 for water/butanol separation [221]. COF membranes fabricated at room temperature via the interfacial polymerization strategy suffered from low crystallinity. The low crystallinity led to truncated selectivity and permeability; thus, the advancement of synthetic strategies is essential. The crystallinity of the COF membrane influenced by the reversibility and robustness of COF linkage (bond strength), reaction rate, and the synthetic condition (high temperature, catalyst, concentration) initiated reversible condensation reactions. For instance, Banerjee and co-workers reported the in-situ growth of crystalline COF-based membranes [222]. The aromatic diamine co-reagent (PTSA⸳H_2_O) was mixed with water to form salt. The resultant organic salt and 1,3,5-triformylphloroglucinol were shaken to produce a dough followed by a knife-cast on a plate to fabricate film. Lastly, baking the film at elevated temperatures (60–120 °C) for 12–72 h led to the in situ growth of the COF membrane. This strategy extended to synthesize TpBD(Me)_2_-, TpAzo-, TpBpy-, TpOMe-Pa1-, TpOMe-BD(NO_2_)_2_-, TpOMe-Azo-, and TpOMe-Bpy-based COF membranes [223,224]. Notably, the M-TpTD COF-based membrane displayed an acetonitrile flux of 260 Lm^−2^h^−1^bar^−1^. In addition to the liquid–liquid interface, solid–vapor or liquid–vapor interfaces for COF membrane fabrications required high temperatures [26,225,226]. The interfacial polymerization of 1,3,5-triformylphloroglucinol and 1,4-phenylenediamine (vapor phase) in the presence of n-octanoic acid afforded crystalline and porous TFP-PDA COF membranes with a thickness of 120 nm. Notably, the TFP-PDA membrane exhibited an ultrahigh permeance towards water (411 Lm^−2^h^−1^bar^−1^) and acetonitrile (583 Lm^−2^h^−1^bar^−1^). These varying synthetic strategies were used to compare the advantages and disadvantages of constructing frameworks (Table 3). Notably, COF synthesis in ambient conditions elevated the interdisciplinary research to address other challenges. For instance, room-temperature synthetic methodology creates biomolecule building blocks that are unstable at higher temperatures. Despite the simplicity, the prepared frameworks in ambient conditions exhibited stability in harsh mediums and displayed a high degree of porosity and crystallinity.

## 6. Structural Analysis and Characterization

COFs synthesized by stitching organic building blocks using a range of coupling reactions are typically characterized by various physiochemical techniques to establish structural and physical properties. In this section, we describe the analytical techniques to comprehend the structural, physical, and chemical features of porous frameworks. 

### 6.1. Powder X-ray Diffraction and Crystallography 

Powder X-ray diffraction (PXRD) patterns with distinct and clear diffraction signals helped us to evaluate the structure and crystallinity of the framework. Structural simulations together with calculated PXRD comparisons with experimental PXRD patterns aided the correct prediction of COF structures. To further evaluate the stacking layer patterns (AA or AB stackings) of 2D COFs, density functional tight binding (DFTB) calculations along with optimization of conformation was pivotal [227,228]. In addition to eclipsed and staggered modes, the slipped AA-stacking mode was also observed, which, in turn, underlined the roles of topology, planarity, bulkiness, and dynamic linkages. It is worth understanding that stacking energy differs drastically as the stacking pattern changes. Generally, the AB-staggered mode has reduced π–π interlayer interactions resulting in low stabilization energy as compared to the AA-staggered mode, thereby influencing PXRD profiles. In addition to valuable comparisons between calculated and experimental PXRD patterns, the Pawley refinement offers a measure to justify the space groups of COFs, which is reflected in the parameters R_wp_ and R_p_. In addition to PXRD, small-angle and wide-angle scattering values (SAXS/WAWS) reflect the extent of the polymerization and crystallinity of COF suspensions [229,230].

The influence of temperature and pressure on COF structural changes are usually investigated via in situ XRD. To reveal the thermal and pressure stability values, the diffraction intensity against pressure or heat underlined the structural information. High temperature favored a change in the crystalline structure of the framework, whereas high pressure was unfavorable to crystalline COFs. Three-dimensional imine-based COF-300, COF-303, LZU-79, and LZU-111 were synthesized as single crystals. These COF crystals were resolved using single-crystal X-ray diffraction (SXRD) to generate detailed crystal parameters, including atomic positions, bond lengths, bond angles, and unit cell parameters [231]. This comprehensive crystal structure analysis is still in its infancy; however, it offers a method to understand host–guest interactions and mechanistic studies.

### 6.2. Porosity 

Both 2D and 3D COFs should be appropriately activated before understanding the textural parameters. In general, COFs are usually activated by solvent exchange (low-surface-tension solvents), super critical drying, Soxhlet extraction, and vacuum drying, followed by N_2_ adsorption isotherm at 77 K to obtain pore volume, pore size, and surface area. The physisorption isotherms of the framework are divided into six types of micropores, mesopores, and macropores. More importantly, the number of COFs is of significant importance; in general, the weight of the tested COF multiplied by specific surface area (m^2^/g) should be equal to 100 m^2^ or greater. 

### 6.3. Thermal Stability and Chemical Stability

Thermogravimetric analysis (TGA) measures the mass loss versus temperature in a controlled atmosphere. This helps us to understand the thermal stability of frameworks. Moreover, the frameworks first undergo a loss of solvent molecules within a temperature range of 60–120 °C, followed by the structural modification or collapse of frameworks (350–450 °C). Furthermore, TGA measurements as mass loss are not inevitably associated with structural change; therefore, the measurement should be complementary to variable temperature powder X-ray diffraction (VT-PXRD) and in situ PXRD. Notably, TGA measurements can be used to roughly estimate the pore volume. The mass loss ascribed to the release of the trapped solvent in the pores near the boiling point can reflect the pore volume of the COFs. In addition to thermal stability, the chemical stability of COFs is fundamentally important to tune the pore environment via established principles of organic and organometallic chemistry. To evaluate the frameworks’ chemical stability, COFs were immersed in acid (diluted and concentrated hydrochloric acid), base (sodium hydroxide), protic (water, ethanol), and aprotic solvents (tetrahydrofuran, dichloromethane, acetone) for multiple days to weeks. The physiochemical analysis comparison of pristine and immersed frameworks underlined structural integrity, porosity, crystallinity, and morphology, which is fundamental to develop scaffolds such as novel sensors and catalytic systems. For instance, Py-Azine COFs exhibit excellent chemical stability in 1M HCl and 1M NaOH [106]. In addition, TpOMe-BD(NO_2_)_2_, TpOMe-Azo, and TpOMe-Bpy with a 2,4,6-trimethoxy-1,3,5-benzenetricarbaldehyde group displayed high chemical stability outcomes in H_2_SO_4_ (18M), HCl (12M), NaOH (9M), boiling water, and common organic solvents [224]. 

### 6.4. ^13^C NMR and Fourier-Transform Infrared Spectroscopy

^13^C CP-MAS NMR and FT-IR spectroscopy bolstered the successful synthesis, purity, and local chemical environment of the framework. This reinforced the chemical state of multiple functionalities and identities, for instance, dynamic imine-linked COFs showed the appearance of -C=N functionalities at 159 ppm in ^13^C CP-MAS NMR and at 1620 cm^−1^ in FT-IR spectroscopy with a disappearance of -C=O and -NH_2_ moieties. In addition, FT-IR spectra showed absorption values at 1774 cm^−1^ and 1720 cm^−1^ corresponding to the asymmetric and symmetric vibrations, respectively, of the -C=O groups of five-membered imide rings. Moreover, ^13^C CP-MAS NMR exhibited the carbonyl carbon of an imide ring at 163.0–165.0 ppm. 

### 6.5. Morphology 

Scanning electron microscopy (SEM) and transmission electron microscopy (TEM) are useful to investigate the morphology, elemental composition, and particle size of frameworks. To obtain high-quality SEM images, sputter coating can be performed by applying an ultra-thin coating of electrically conductive metal, such as gold. These microscopies can be combined with energy dispersive spectroscopy (EDS) or energy dispersive X-ray analysis (EDX) to assist elemental quantitative and qualitative composition determinations in COFs. This technique is particularly important to study the presence of immobilized transition metals in COFs via pore surface engineering, which is fundamental to characterize heterogeneous catalysts. In addition, high-resolution TEM allows the visualization of structural features using the low-dose TEM technique based on the use of a direct-detection electron-counting camera. For instance, Peng et al. reported the honeycomb-like porous structure of a TPA-COF nanosheet using high-resolution TEM. The high-resolution TEM image displayed hexagonally arranged white contrasts surrounded by six black dots, which corresponded to a 1D pore channel and building blocks [232]. Furthermore, Li et al. exhibited the honeycomb-like porous structure of COF-1 along with the [111] direction with a pore opening of 3.0 ± 0.2 nm using a low-dose TEM technique. Notably, the easy structural damage of COFs under electron beams created novel challenges to produce magnified-resolution images [233]. 

## 7. Growth Mechanisms of COFs

Considerable progress has been achieved in understanding the growth mechanisms of COFs from organic building blocks. The amorphous-to-crystalline transformation mechanism was highlighted by Dichtel and co-workers. The robust and rigid monomer units in solution after the addition of acid catalyst rapidly produced a solid product, which, in most cases, was an amorphous polymer. The solid amorphous product at elevated temperatures under a sealed condition converted kinetically generated amorphous product into thermodynamically stable crystalline networks. For instance, the growth mechanism process of the TAPD-PDA COF was studied under two isolated conditions. First, the initial polymer was characterized by PXRD, underlining the amorphous nature. Thereafter, the amorphous product was re-subjected to the optimized COF synthetic condition (70 °C) without any addition of solvent, and monomers exhibited intense diffraction peaks, underlining the crystalline nature of frameworks. Second, the solid amorphous product without any addition of catalysts of specific concentrations displayed an absence of intense diffraction peaks, thereby confirming an amorphous-to-COF transformation. Notably, the transformation process did not show any major change in morphology. Furthermore, the initial TAPB-PDA amorphous product exhibited a surface area of 18 m^2^/g and experience exponential increase in the crystalline TAPB-PDA framework (287 m^2^/g) after being subjected to two days of optimized reaction conditions [234]. The research report by Gao et al. and the Guo research group for the synthesis of imine-linked COF-1 and β-ketoenamine TpBD COF also demonstrated a reformation mechanism [235,236]. In addition to the amorphous-to-crystalline transformation, Liu and co-workers illustrated a dissolution–recrystallization mechanism using 2,6-dihydroxynaphthalene-1,5-dicarbaldehyde, and 2,4,6-tris(4-aminophenyl)pyridine building blocks, which was similar to the amorphous-to-crystallization transformation mechanism with one observed difference in the change in morphology during the growth process. First, the initial solid precipitate showed irregular nanoparticle features followed by an increment in the reaction time creating a microsphere-to-uniform-microfiber transformation. Finally, the process of amorphous-to-crystalline transformation showed intense peaks in the PXRD profile [237].

**Figure 9:**
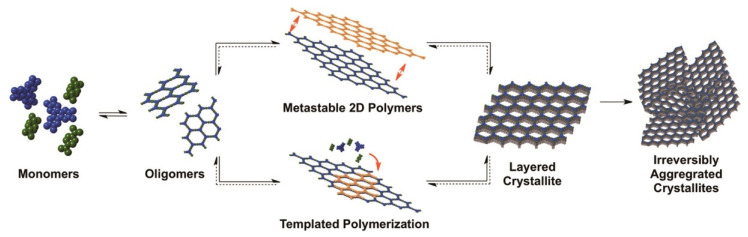
Proposed models of boronate ester COF-5 growth. Reproduced with permission from ref. [238] (Copyright © American Chemical Society).

The reaction-induced mechanism was entirely different from amorphous-to-crystalline and dissolution–recrystallization mechanisms due to no amorphous polymer formation in the early stage of the reaction. Dichtel and co-workers first investigated this mechanism using boron-containing COF-5 [238] (Figure 9). In the initial stage, building blocks condensed into soluble oligomers, which, upon nucleation, afforded crystallites aggregates. Notably, it was observed that precipitates collected after five minutes displayed identical PXRD profiles to COF-5, with no evidence of a co-crystallized monomer units or any impurities. This ordered crystalline network of initially formed solid products was the differentiating point of two other mechanisms, which usually formed amorphous solid products. Furthermore, Bredas et al. illustrated two pathways of COF-5 nucleation and growth processes, i.e., the lateral growth of stacked structures and stacking between large oligomers using the kinetic Monte Carlo simulation [239]. In addition, attempts were made to investigate the organic linker exchange of stable dynamic-linked COFs. For instance, Zhao et al. [240] reported the introduction of benzidine at elevated temperatures to an imine precursor synthesized by a reaction of 1,3,5-triformylphloroglucino and mono-functional amine. The PXRD profile displayed crystallinity and higher porosity in comparison to the framework synthesized by the solvothermal process. A similar strategy was employed by Miao et al. for the construction of crystalline frameworks from an amorphous covalent organic polymer [241]. Covalent organic polymer synthesized from 1,4-phthaldehyde and 1,3,5-tris(4-amidophenyl)triazine underwent ligand exchange using different building blocks; 2,5-dihydroxyterephthaldehyde, 2,5-dimethoxyterephthaldehyde, 2,3-dihydroxyterephthaldehyde, and 2,3-dimethoxyterephthaldehyde afforded COF 1-4. This one-step direct synthesis method developed our understanding of the mechanistic viewpoint and expanded the scope of crystalline COFs. Yaghi and co-workers highlighted the COF-to-COF conversion protocol for linker substitution, cyclization, and oxidation. Imine-linked ILCOF-1 based on 1,3,6,8-tetrakis(4-formylphenyl)pyrene and1,4-phenylenediamine converted to organic linker (2,5-diaminobenzene-1,4-dithioldihydrochloride and 2,5-diaminobenzene-1,4-dihydroxydihydrochloride) substitution and oxidative cyclization afforded two isostructural COF-921 and LZU-192. Both COF-921 and LZU-192 displayed good thermal stability (400 °C) and a higher degree of crystallinity. Furthermore, the frameworks exhibited a surface area and pore size larger than 1500 m^2^/g and 20 Å, respectively [143].

## 8. Scalability Challenge

The limited scalability and fragility components are critical challenges in the advancement of COFs towards commercialization and industrial applications. The stable dynamic linkage is essential to connect building blocks in a network structure, which usually require closed conditions to maintain the reversibility of condensation reactions. This is usually achieved by sealed Pyrex tubes with low internal pressures or a hydrothermal reactor. The low internal pressure inside the Pyrex tube permits the slow diffusion of water to enable nucleation and crystallization. In contrast, heated building blocks at elevated temperatures in an open atmosphere can disturb reversibility via a loss of water molecules, thereby exhibiting a poor structural order. Consequently, it is difficult to maintain an ideal reaction condition to synthesize COFs on a larger scale, thereby the percentage yield of most COFs is in the order of milligrams. In addition, a prolonged reaction time, high-temperature boiling solvents, and activation prohibit COFs’ development in the interdisciplinary research.

### 8.1. Synthetic and Crystallization Aspects

COFs’ high degree of crystallinity differentiates the porous organic material from conventional amorphous polymers. The entire organic character of COFs presents their fundamental role and the utilization of robust dynamic coupling reactions to impart chemical/thermal stability. For example, β-ketoenamine and benzoxazole COFs showed tolerance towards acidic and basic conditions [242]. The high network order in COFs arises due to reversible bond formations, which permits self-corrections to minimize defects and circumvents the formation of amorphous products. This reversibility induces the error-correction mechanism, where building blocks oriented in a specific direction within the growing network balance the kinetics to obtain an ordered structure. For instance, the amorphous-to-crystalline transition in imine-linked 2D COFs and building block exchange confirms the potent role of the error-correction mechanism [234,243]. Furthermore, the slow growth of 3D imine-linked COF single crystals in the presence of competitive reagent. These mechanistic studies foreshadowed the advancements in synthetic strategies of COFs; however, a large gap still remains regarding large-scale COF production. One of the prime issues highlight the time required for reversibility-induced error corrections. Additionally, the stability of dynamic linkages significantly extends the crystallization timeline. For instance, imine-linked, β-ketoenamine-linked, and other COFs required two to seven days to synthesize crystalline frameworks [102]. As a result, it is inevitable to invent novel synthetic strategies to minimize lower degrees of long-range orders and reduce error-correction durations. The novel synthetic methods for COFs’ large-scale syntheses address the challenges and limitations directly associated with the reversibility-induced error corrections. Two-dimensional crystalline COFs obtained by the slow addition of building blocks and reagents tuned the reaction equilibrium and facilitated error corrections in a closed system (Pyrex tube, low internal pressure = 150 mTorr) as per the Le Châtelier’s principle. Furthermore, this one-pot multistep process may lead to the involvement of side products. This is a common practice at the lab scale; however, an imitation of this approach on a large scale is dubious.

The addition of catalysts is highly relevant to hasten COFs reaction rates. However, an aspect of green chemistry is equally relevant. The stitching of building blocks via ultra-stable dynamic linkages is scarcely practicable without a catalytic mediation. Acetic acid is one of the most widely used catalysts for COF synthesis. Brønsted acids, such as H_3_PO_4_, HCl, and CF_3_SO_3_H, displayed poor crystallinity and porosity properties due to the strong binding with aromatic diamine, which in turn negatively influenced the nucleophilicity and acid–amine proton transfer reaction required for COF synthesis. Moving beyond the acid catalyst, Lewis acid catalysis for a transamination reaction drastically reduced the reaction time and temperature, however, it introduced activation challenges. In addition, the nucleophilic catalyst and ionic liquids positively influenced the reaction rate. The generalization and widespread usage of ionic liquid in COF synthesis is a significant challenge to expand the library of frameworks in ambient conditions. The sustainability of the catalysts has yet to be considered to a large extent. Nonetheless, under the principle of green chemistry, it is conceivable that toxicity, efficacy, aquatic damage, and safe handling will emerge as relevant factors during synthetic expansion. Apart from the mechanisms and green chemistry, stable linkages are indispensable to avert the initiation of the hydrolytic decomposition of COFs. This primarily depends on the choice of building blocks and dynamic linkage. In general, an electron-withdrawing group directly attached to imine nitrogen exponentially decreases the nucleophilicity of imine nitrogen makes proton attack unfavorable. Consequently, β-ketoenamine, hydrazone, and azine linkages exhibited extra-chemical and thermal stability; however, limited building blocks do not help in the design of stable COFs. It is important to discover novel stable linkages to synthesize COFs at ambient temperature conditions. Furthermore, the robust interlayer stacking interactions bolster addition stability. The electrostatic repulsion among polarized bonds and π–π stacking interactions between an individual layers improves stability. Notably, the presence of intramolecular hydrogen bonding interaction via the uniform distribution of hydroxy group, methoxy groups significantly improve crystallinity, porosity, and chemical stability of COFs [138,140]. Therefore, an effective, economical, scalable, and environment friendly synthetic methodology is desirable to fabricate stable COFs.

Apart from synthetic challenges, the crystallization of extended structures is relatively difficult as compared to discrete structures. The crystallization process requires reversible bond formation to allow the self-correction of defects and avoid the design of an amorphous network. This involves the judicious design of building blocks and the specific positioning of functionalities to add covalent linkages between organic molecules. In addition to the building blocks’ symmetricity, the appropriate stoichiometric quantities of building blocks are important to control the equilibrium in a closed system. The combination of hydrophilic and hydrophobic solvents (*o*-dichlorobenzene/*n*-butanol, mesitylene/1,4-dioxane) further facilitates water partition between a reaction mixture, which is effective in reversibly controlled reaction rates. COFs constructed through an irreversible coupling reaction are pivotal to address crystallization. The solvent trapped in the pores adds activation issues and differentiates frameworks as ultra-stable and fragile COFs.

### 8.2. Activation Aspect 

During COF synthesis, solvent molecules, solvated counterions, and building blocks are trapped in the pores of the frameworks. The necessity to access the permanent porosity for an ingress of foreign functionalities permits various chemical transformations. In order to tune the chemical and steric environment of COFs, the trapped solvent or monomer molecules must be removed―a process that is called activation. This task is more stimulating due to the insolubility of COFs. The most common activation techniques include solvent exchange, vacuum drying, and supercritical CO_2_ drying. 

#### 8.2.1. Solvent Exchange and Vacuum Drying 

**Figure 10:**
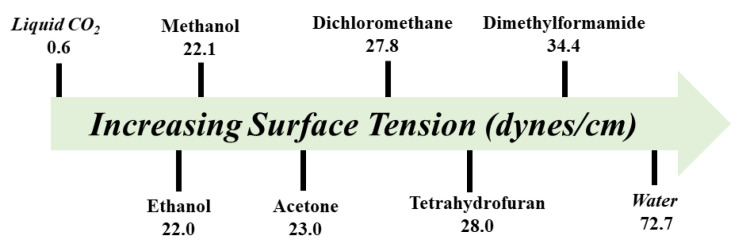
Surface tension of the most common organic solvents for COF activation.

In most instances, the as-synthesized COFs, heated (60 °C–80 °C) under vacuum, may be sufficient to remove the trapped solvent. This is observed in an ultra-robust imine-linked, β-ketoenamine-linked, hydrazone-linked, azine-linked, imide-linked, and other COFs. On the contrary, fragile COFs collapse under vacuum due to high surface tension and capillary forces imposed on the structure by the liquid-to-gas phase transformation of trapped solvent molecules (Figure 10). One of the plausible methods is to exchange the solvent with a lower boiling-point and lower-surface-tension solvent prior to heating the sample under vacuum. For instance, the N_2_ adsorption desorption isotherms for TAPB-PDA COF activated directly from perfluorohexane and hexane exhibited BET surface areas of 2121 m^2^/g and 2060 m^2^/g, respectively, compared to TAPB-PDA COF activated from methanol (173 m^2^/g), acetone (91 m^2^/g), tetrahydrofuran (28 m^2^/g), dioxane (113 m^2^/g), and dimethylformamide (172 m^2^/g) [244]. To appropriately accomplish solvent exchange, COFs should be washed carefully with the reaction solvent to exclude either impurities or unreacted building blocks followed by COFs soaked in low-boiling-point and low-surface-tension solvent to ensure complete infiltration inside the pores. This process requires a prolonged amount of time to make sure the fresh solvent penetrates into the pores of COFs. Once the solvent exchange is complete, heating under vacuum can be applied to ensure complete the activation of the sample. Notably, the heating temperature should be above the solvent boiling point, but below the decomposition temperature of the framework, as obtained by TGA analysis. 

In another work, Verduzco and co-workers elaborate an insight into the relationship between structural integrity and pore size, pore functionality, and pore architecture [245]. Rhombic Py-1P COF with a small pore size (1.5 nm) displayed high crystallinity and porosity after activation with solvents of varying surface tensions, methanol, tetrahydrofuran, and perfluorohexane. Moreover, rhombic Py-2P COF with a larger pore size (2.6 nm) exhibited excellent crystallinity and surface area after activation with methanol or perfluorohexane (3121 m^2^/g and 3312 m^2^/g, respectively), whereas activation with tetrahydrofuran disrupted crystallinity and porosity (91 m^2^/g). Notably, hydroxyl-functionalized rhombic Py-OH1P COF displayed structural robustness, crystallinity, and porosity after activation with methanol, tetrahydrofuran, and perfluorohexane. This underlined the profound role of hydrogen bonding in maintaining the structural integrity of frameworks. A similar activation effect was unambiguously observed for imine-linked COFs of varying pore sizes, TAPB-BTCA (0.9 nm), TAPB-TFPA (1.7 nm), TAPB-TFPB (2.0 nm), and TAPB-PDA (3.3 nm), and pore functionalities, TAPB-FPDA, TAPB-OH PDA, and TAPB-Br PDA. In addition, Soxhlet extraction is another important means for solvent exchange, particularly in the case of linkers, which are difficult to separate due to poor solubility and the solvent being difficult to remove from the pores. For instance, COF-ETTA-EDDA, COF-ETTA-DMDA, TPAPC-COF, and others activated by Soxhlet extraction using anhydrous tetrahydrofuran and acetone displayed significant improvement in crystallinity and porosity [175]. 

#### 8.2.2. Supercritical Drying

Supercritical carbon dioxide (scCO_2_, surface tension = 0.6 dynes/cm) is a milder activation technique especially used for fragile framework in which conventional solvent exchange is unsuccessful and causes structure collapse. Using scCO_2_, the activation process avoids the liquid-to-gas phase transformation of the guest solvent and instead experiences a supercritical phase, thereby eliminating capillary forces and the surface tension effect. From an experimental perspective, the solvated COFs can be placed in a scCO_2_ dryer and cooled to 2–10 °C, followed by exchange with CO_2_. The sample chamber temperature should not drop below 0 °C and fresh CO_2_ can be purged every hour. After multiple exchanges (4–5 times), the sample can be heated to 31 °C and 73 atm, the supercritical temperature and pressure of CO_2_ to release gaseous CO_2_, respectively, often called as bleeding. The activation challenge associated with a fragile framework can be resolute to access porosity. For instance, Medina and co-workers underlined scCO_2_ activation to access the porosity of TA TAPB-COF, TT TAPB-COF, and BDT TAPB-COF fragile COFs with BET surface areas of over 1000 m^2^/g [246]. On the contrary, the extent of crystallinity and porosity decreases using 1,4-dioxane, toluene, and acetone. Additionally, scCO_2_ activated hydrazone-linked TFPB-DHz COF, TFPT-DHz COF, and Py-DHz COF exhibited excellent crystallinity and porosity [247]. The BET surface areas of TFPT-DHz COF, TFPB-DHz COF, and Py-DHz COF were found to be 1199 m^2^/g, 790 m^2^/g, and 932 m^2^/g respectively, while the conventional solvent-activated COFs appeared to be amorphous. Recently, Feriante et al. highlighted the minimization of capillary strain to avoid pore collapse using scCO_2_ and to bolster the rapid synthesis of ultra-robust imine-linked TAPB-PDA, TAPB-OHPDA, TAPB-OMePDA, TAPPy-PDA, and TAPPy-NDI-DA COFs in four hours [248]. The surface area of TAPB-PDA COF after scCO_2_ activation is approximately ten times higher as compared to vacuum activation. In contrast to TAPB-PDA COF, the influence of vacuum or scCO_2_ activation on TAPB-OMePDA and TAPPy-PDA COFs is less noticeable, thereby approving the stabilizing effect due to docking behavior.

### 8.3. Mobile Robotics 

The synthetic development of COFs can benefit greatly by establishing manufacturing equipment. A mobile robot is widely used in the industry to exponentially increase the productivity. The robot has human-like dimensions and can operate to dispense insoluble and soluble solutions with high accuracy and repeatability. Furthermore, the robot arm and the mobile base comply with safety standards. To date, no approaches have integrated COFs with mobile robotics to shorten the timeline for either the synthesis or optimization of reaction conditions. Cooper’s research group recently reported a mobile robot for improved photocatalysts for hydrogen production from water [249]. Notably, the robot performed 688 experiments within a ten-variable experimental space over eight days. This bolstered and shortened the screening timeline to discover an ideal reaction condition for COF synthesis with a high degree of crystallinity and porosity. For instance, Ma’s research group reported the screening of multiple reaction conditions for TPAPC-COF synthesis. The nine reaction conditions were composed of different ratios of solvents, building blocks’ stoichiometry, and activation strategies. The solvothermal reaction of 5,10,15-tris(*p*-aminophenyl)corrole and terephthaldehyde in mesitylene/*n*-butanol at 120 °C exhibited good crystallinity and a BET surface of 745 m^2^/g. However, the similar stoichiometric ratio of building blocks in either ethanol/mesitylene or butanol/*o*-dichlorobenzene displayed lower BET surface areas [250]. This laborious work can be curtailed by assimilating COFs with robotics. In another work, the ideal hydrophobic/hydrophilic solvent combination and catalyst concentration for successful Schiff-base condensation reaction to synthesizing TAT-COF-1 and TAT-COF-2 were reported by Zheng and co-workers. Seventeen reaction conditions comprised varying precursor ratios, catalyst concentrations, and solvents correlated with PXRD profile [251]. The ideal synthetic condition is central to obtain high degree of crystallinity and porosity. Notably, TAT COFs displayed moderate-to-good hydrogen and carbon dioxide adsorption.

Recently, Beuerle, Würthner and co-workers reported the influence of varying reaction conditions to fabricate crystalline COFs from amorphous polymers. The solvothermal reaction of ruthenium(2,2′-bipyridine-6,6′-dicarboxylate)dialdehyde and tetra-(4-anilyl)methane at 60 °C in N,N- dimethylacetamide/mesitylene (1:1) displayed high crystallinity; however, nine solvothermal reactions in one solvent (CH_3_OH, THF, DMAc, DMF, 1,4-dioxane, and dimethylsulfoxide) or a mixture of solvents (DMAc/methanol, DMAc/1,4-dioxane, and DMAc/Mes) at varying temperatures (60 °C and 120 °C) and times (72 h and 96 h) showed poor crystallinity or no solid product [252]. This substantial challenge of optimization for prolonged reactions can be curtailed by designing a suitable robotic system for COF chemistry. This inevitably generates a library of COFs using novel dynamic linkages and bolsters gram-scale synthesis. To summarize, it is important to uncover the optimal conditions for the gram-scale synthesis of ultra-stable and fragile COFs and design innovative linkages in structures. Still, it will be stimulating and offer great intellectual challenges to link synthetic techniques and activation instruments.

## 9. Conclusions, Outlook, and Summary 

Covalent organic frameworks, a novel porous organic material, has been broadly examined in various fields and displays an imperative role in nanotechnology. COFs’ porosity and crystallinity confirms the unambiguous validation of their structures constructed by dynamic covalent linkages using the principle of reticular chemistry. Dynamic covalent chemistry proposes distinct properties, such as thermal stability, chemical stability, and high porosity, of the designed frameworks, which is important in industrial applications. Furthermore, the library of symmetrical building blocks bearing multiple binding sites at a specific position shows the structural and topological diversity of COFs. This expansion of organic chemistry to an extended network was comprehensively discussed in this review. The design principle can be useful in building either reported or hypothesized structures with varying pore shapes and sizes. This variable pore geometry offers ample opportunities to create an ideal electronic and steric environment either via pore surface engineering or de novo approaches. With these accomplishments, COFs have a unique position in the fields of material sciences and chemistry. However, despite their many favorable characteristics and great advances, the fields require considerable research to urgently address the level of synthetic control, from molecular geometry to network geometry. This requires manipulation at the fundamental level, including synthetic, crystallization, activation, controlled morphology, and coupling chemistry, which hinder the large-scale synthesis of COFs. The review underlined these issues, which deserve attention in future research.

1. The building blocks for constructing COFs are either unavailable commercially or expensive. The most common starting materials involve multistep tedious reaction under severe conditions, and the complicated purification step hampers gram-scale synthesis. In addition, the role of the catalyst in COF synthesis remains a nascent field from a mechanistic perspective, which requires in-depth investigation, especially when moving from protic acid to metal salt. The stitching of building blocks in the presence of a novel catalyst may provide a plausible solution to poor-crystalline COFs and extends the reaction time, but limits safety and presents sustainability concerns. 

2. The uniformity and reproducibility in the large-scale synthesis of crystalline and porous COFs are central to commercialization. However, the complexity in structural, activation, and crystallization aspects at the milligram scale demands sustainable tactics for scalable production and batch-to-batch reproducibility. The solvent-free mechanosynthesis might be a plausible method, but suffer from a limited scope. It is imperative to focus on irreversible coupling reactions to develop a simple and effective method for well-defined, uniform, and reproducible COFs, which is still in its infancy. 

3. The scalable and sustainable preparation of COFs will significantly be assisted by using manufacturing equipment. The use of automation and robotics can not only bolster the optimization of reaction conditions to fabricate established or novel linkages in a short time, but also positively influence large-scale synthesis at the industrial level for wider applications. Thus, this is only the beginning of this exciting field. 

## Figures and Tables

**Table 1:** Previous reviews of covalent organic frameworks (COFs).

Categories	Main Contents
Comprehensive Reviews	Structure, synthesis, and applications [47,48,49,50,51]
Structures	Linkages: Boron chemistry and beyond [14]; Schiff-based and imine-based COFs [52,53]; triazine networks [54,55,56,57].
	Architectures: Structural design and principles [7,37].
	Monomers: Porphyrin and phthalocyanine COFs [58]; boronic-acid-based COFs [59,60].
	Pore shape: Multi-porous COFs [61] and pore surface engineering [16,17,18,42].
Applications	Catalysis: Single-site catalysis [62]; heterogeneous catalysis [19,20,21,63,64]; electro- and photocatalysts [28,29,65,66,67,68,69,70,71] Sensing and analysis: Chemical sensing [43,72,73,74,75,76]; analytical progress [77,78,79]; optical and electronic applications [29,35,80,81,82].
	Biomedical: Nanomedicine and drug delivery [31,32,33,83,84,85,86]. Absorption and storage: Gas storage [87]; hydrogen storage [88,89]; CO_2_ capture [90,91,92]; energy storage [28,30,82,93]. An overview: various applications [44,47,49,50,51,54,94,95,96,97,98,99].
Synthetic Methods	Solvothermal and alternative synthetic methods [100,101,102,103].

**Table 2:** Characteristics of the most widely used dynamic linkages for COFs.

Bonds	Dynamic Linkages	Characteristics
B―O	Boronate Ester	Excellent crystallinity and thermal stability (600 °C), but sensitive to water, acid, and base.
	Boroxine	Excellent crystallinity and thermal stability (500 °C), but sensitive to water, acid, and base.
C―N	Imine	Good crystallinity and excellent thermal (500 °C) and chemical stability.
	Hydrazone Imide	Good crystallinity and thermal stability (300 °C), but better chemical stability as compared to imine linkages.
		Good crystallinity and chemical stability, but excellent thermal stability (500 °C).
	β-ketoenamine	Moderate crystallinity and good thermal stability (300 °C). Excellent chemical stability even in acid (HCl), base (NaOH), and boiling water.
	Azine	Moderate crystallinity and thermal stability (250 °C). Excellent chemical stability even in acid (HCl), base (NaOH), water, and organic solvents.
	Squaraine	Moderate crystallinity and good thermal stability (300 °C). Excellent chemical stability even in acid (HCl), base (NaOH), water, and organic solvents.
	Triazine	Poor crystallinity and good thermal (400 °C) and chemical stability.
	Nitrone	Good crystallinity. No precise description on thermal and chemical stability.
C―C	Alkene	Moderate crystallinity and good thermal (350 °C) and chemical stability.
C―O	Ester	Good crystallinity. No precise description on thermal and chemical stability.
	1,4-dioxin	Good crystallinity and thermal stability (400 °C). Good chemical stability in both acid (HCl) and base (NaOH).

**Table 3:** Comparison between different synthetic methods.

Synthetic Method	Advantages	Disadvantages
Solvothermal	Widely-used method for range of monomers and some COFs can be synthesized on a large scale.	Time-consuming (3–7 days), flame torch, and requires high temperatures.
Ionothermal	Promotes green chemistry. Molten salts are used as solvents and catalysts.	Lacks long-range order in framework (amorphous) and requires high temperatures.
Microwave	Lower reaction time and uses an alternative source of energy. Fast and cleaner products.	Requires high temperatures in some cases.
Sonochemical	Rapid, cost-effective, and bolsters green chemistry.	Requires high-temperature conditions and hinders large-scale synthesis.
Mechanochemical	Solvent-free, room temperature, economical, and environmentally friendly. Simple manual grinding is required.	Suffers from diversity of building blocks and dynamic linkages.
Room temperature	Simple, easy, and promotes green chemistry.	Restricted to a few building blocks.
